# Identification of potential microRNA panels for pancreatic cancer diagnosis using microarray datasets and bioinformatics methods

**DOI:** 10.1038/s41598-020-64569-1

**Published:** 2020-05-05

**Authors:** Roshanak Shams, Samaneh Saberi, Mohammadreza Zali, Amir Sadeghi, Soudeh Ghafouri-Fard, Hamid Asadzadeh Aghdaei

**Affiliations:** 1 Research Center for Gastroenterology and Liver Disease, Shahid Beheshti University of Medical Sciences, Tehran, Iran; 2 Department of Medical Genetics, Shahid Beheshti University of Medical Sciences, Tehran, Iran; 3 HPGC Research Group, Medical Biotechnology Department, Biotechnology Research Center, Pasteur Institute of Iran, Tehran, Iran

**Keywords:** Pancreatic cancer, Gene expression, Computational models, Functional clustering, Gene ontology, High-throughput screening, Microarrays

## Abstract

Pancreatic cancer (PC) is a malignancy with little/no warning signs before the disease reaches its ultimate stages. Currently early detection of PC is very difficult because most patients have non-specific symptoms leading to postponing the correct diagnosis. In this study, using multiple bioinformatics tools, we integrated various serum expression profiles of miRNAs to find the most significant miRNA signatures helpful in diagnosis of PC and constructed novel miRNA diagnosis models for PC. Altogether, 27 differentially expressed miRNAs (DEMs) showed area under curve (AUC) score >80%. The most promising miRNAs, miR-1469 and miR-4530, were individually able to distinguish two groups with the highest specificity and sensitivity. By using multivariate cox regression analyses, 5 diagnostic models consisting of different combinations of miRNAs, based on their significant expression algorithms and functional properties were introduced. The correlation model consisting of miR-125a-3p, miR-5100 and miR-642b-3p was the most promising model in the diagnosis of PC patients from healthy controls with an AUC of 0.95, Sensitivity 0.98 and Specificity 0.97. Validation analysis was conducted for considered miRNAs on a final cohort consist of the microarray data from two other datasets (GSE112264 & GSE124158) . These results provide some potential biomarkers for PC diagnosis after testing in large case-control and cohort studies.

## Introduction

According to GLOBOCAN 2018, pancreatic cancer (PC) is the 4th leading cause of cancer-related death and is associated with high mortality and poor prognosis^1^. PC is a malignant condition with little/no warning signs before the disease reaches its ultimate stages^2^. The majority of patients with PC are already reached to either locally advanced or metastatic level in the asymptomatic phase before referring to the clinic and as many as 80% are categorized in unresectable group^3^. The average survival rate for the PC is reported to be less than one year. Some studies reported that the 5-year survival rate would increase notably if PC patients were diagnosed at initial stages and subjected to surgical resection followed by chemotherapy^4,5^. Currently early detection of PC is very difficult because most patients are found with non-specific symptoms leading to postponing the correct diagnosis^6^. On the other hand, sometimes pancreatic mass is indistinguishable from chronic pancreatitis or benign pancreatic cysts, so the results of pathological assessment of biopsies obtained from the lesion could be not informative^7^. Moreover, cytological analysis of the sample taken by endoscopic ultrasound-guided fine needle aspiration (EUS-FNA) may be non-precise because of sampling complications, inflammation coexistence or other conditions^8^. Therefore, finding possible non-invasive biomarkers at early stages of PC progression is crucial for evaluation of high-risk subjects to establish follow-up strategies and surgical resection of primary malignancy. In this regard, numerous scientists tend to identify biomarkers that could help gastroenterologists and pathologists in PC detection, and finding potential biomarkers with the possibility to be accessible in a less invasive method have become a research trend. The ultimate biomarkers must be easily detectable with fine sensitivity and specificity and also must discriminate PC from other benign pancreatic diseases^9^. Blood is a simply reachable and rather steady sample to find alerting biomarkers. Technological advances in the recent years have provided possibilities to detect circulating biomarkers based on “omics” research, relying on proteins, cell-free DNAs, non-coding RNAs, circulating tumor cells (CTCs), and exosomes molecular contents^10^. MicroRNAs (miRs) are small (~22 nucleotides) non-coding RNAs that have gene regulatory roles via targeting the 3′-untranslated region (3′-UTR) of their target mRNA and finally cause either translational repression or mRNA degradation^11,12^. Up to now, a number of biomarkers have been introduced as PC biomarkers such CEA, CA19-9, CA125 and CA72-4. Nonetheless, none of these tumor markers has shown efficient sensitivity or specificity for diagnosing PC at primary stages and have been used for post resection monitoring rather than earlier detection purposes. MiRNAs seem to be truly stable in blood and several authors reported that miRNAs show dysregulation in pancreatic diseases being able to differentiate PC from pancreatitis, pancreatic benign masses as well as normal subjects^13^. Furthermore, owing to advanced technologies in high-throughput molecular methods the understanding of the pathophysiology of pancreatic cancer have been improved. Various genome-wide mRNA and miRNA expression profiling studies using microarray-based and NGS approaches have provided important insights into the phenotypic characteristics of pancreatic cancer^14^. In this study, using multiple bioinformatics tools, we integrated various serum expression profiles of miRNAs to find the most significant potential miRNA signatures helpful in the diagnosis of PC and constructed a novel miRNA- mRNA regulatory network in PC using bioinformatics approaches. Next, we investigated the molecular mechanisms downstream of the captured miRNA signatures and their predicted target genes correlated to PC progression and analyzed them in a logistic model.

## Material and method

### Microarray datasets search

In order to find proper miRNA expression profiles in microarray datasets, we conducted a systematic search in Gene Expression Omnibus (GEO) database (https://www.ncbi.nlm.nih.gov/geo/)^15^. Using the keywords “Pancreatic cancer” and “Serum”, at the first step we reached 900 datasets. Then, we limited the results using ‘Homo sapiens’ and ‘Non-coding RNA profiling by array’ filters, so we reached to 16 datasets. Finally, by setting the sample count on more than 200 samples, 4 final datasets were achieved.

### Differentially expressed miRNAs (DEMs) detection

The DEMs were obtained using the online tool GEO2R in the GEO database^15^, which makes evaluations using the GEOquery and limma R packages from the Bioconductor project to compare two or more groups of samples in a GEO dataset. Normalization has been carried out using the RMA algorithm. To keep away from the false-positive/negative and the differences between microarray platforms, the microarray gene-expression profiles of PC groups was compared to the normal groups of each dataset separately. |log2 (RMA signal intensity fold change) | ≥1 and p value > 0.01 was set as cut off to identify the significant DEMs. After that, significantly expressed DEMs in each study were listed respectively.

### Combination of the data

A Venn diagram creator tool in Bioinformatics & Evolutionary Genomics source was used to combine all datasets and find the overlapping DEMs (http://bioinformatics.psb.ugent.be/webtools/Venn/). In order to cover more miRNAs with differential expression and to prevent missing of critical genes that may not have shown differences in expression in one study for any reason, it was decided to select the DEMs overlapped between at least 3 of datasets.

### Area under curve (AUC) analysis

The expression values of all overlapping DEMs were extracted and imported to GraphPad Prism software. After normalization of the values, Receiver-operating characteristic (ROC) curves and area under the ROC curve (AUC) were used to assess the detection ability of each miRNA in discriminating PC patients from the control group based on the sensitivity and specificity of each DEM.

### Hierarchical clustering analysis

Individual expression values of significantly up/down regulated DEMs in PC (27 DEMs) were logarithm transformed and were used as input values for the hierarchical clustering algorithm. The following criteria was applied: The distance chose “Pearson Correlation”, and the linkage selected “average”. The result is demonstrated as a Heatmap.

### Expression correlation analysis

The expression values of the DEMs were clustered using the k-means method. The median of expression values of all miRNAs for each sample was used as the representative expression for the cluster. Using R programing software^16^ and the corrplot package^17^, Pearson’s correlation for 27 DEMs expression profiles were analyzed and demonstrated as a Corrplot.

### MiRNA-miRNA interaction network

MiRNet is an online tool suite designed for precise analysis and functional interpretation of miRNAs and xeno-miRNAs^18^. This tool holds numerous high-quality science-base to link miRNAs to their targets and other correlated molecules. Network analysis was carried out using functional annotations based on the Kyoto Encyclopedia of Genes and Genomes (KEGG) pathway database, and by the use of the hypergeometric algorithm for enrichment analysis of loaded data, a table of validated miRNA target genes was achieved using MiRNet.

### MiRNA-mRNA interaction

MirDIP, a miRNA target prediction online tool, supplies almost 152 million human miRNA–target predictions, which were gathered from 30 different resources. It also provides an integrative score, which was statistically concluded from the acquired predictions, and was assigned to each unique miRNA–target interaction to give a unified measure of confidence^19^. Using this tool we reached to predicted target genes lists of each considered miRNA.

### Protein-protein interaction (PPI) network and functional enrichment analysis

Interactions between the target genes of selected miRNAs were predicted using the STRING database v 9.0.5. Confident interaction score was set on ≥0.7. The PPI networks were uploaded and visualized to Cytoscape software^20^ and top modules of PPI network were picked using Molecular Complex Detection (MCODE)^21^ with the inclusion criteria as follow: degree =2, node score =0.2, k-core=2, max. The average degrees of MCODE score and nodes in modules were chosen as the threshold, thus we set MCODE scores ≥9 and hub nodes ≥9 as criteria. Functional enrichment analysis was performed using DAVID for all targets and top modules respectively.

### Multivariate regression analysis

The expression of each miRNA was in continuous format and described as mean and standard deviation (SD). Candidate miRNA expressions were examined on the basis of their univariable association with PC. The full model with the covariate effects was built according to five clusters obtained from bioinformatics analysis, using the stepwise inclusion method. The estimation of the coefficients in each regression model was checked by multicollinearity by analyzing variance inflation factor (VIF). A variable whose VIF values were more than 10 may need further investigation. The significance of covariates in each full model was further tested by the backward elimination method. The akaike information criterion (AIC) and bayesian information criterion (BIC) were computed each time a variable was included/excluded and the model with lower AIC or BIC was preferred. We also determined the performances of each model by examining measures of calibration and discrimination. Calibration points out how nearly the predicted probability of having PC agrees with the observed PC status. This was assessed by Hosmer-Lemeshow test. Discrimination expresses the ability of the clinical decision rule to differentiate between individuals with and without PC. This was assessed by calculating the area under the ROC curve (AUC) statistic. We considered an AUC value of 0.5 as no discrimination, and 1 as perfect discrimination. All analyses were performed by using Stata software (version 14). Results were statistically significant by p < 0.05 levels.

### Validation

As a validation set, an independent cohort composed of serum miRNA expression profiles from patients with pancreatic cancer and healthy controls was provided. The subjects were chosen from two other GEO datasets (GSE112264 & GSE124158) consisting of serum miRNA profiles from PC patients and healthy controls (70 controls and 81 PC). The diagnostic performances of the considered models were checked through determining the combined AUCs for each considered panel. For this aim, the performances of each miRNA individually and also together as panels were analyzed and AUC scores and ROC curves for each model were obtained.

## Results

### Microarray datasets search results

**Figure 1:**
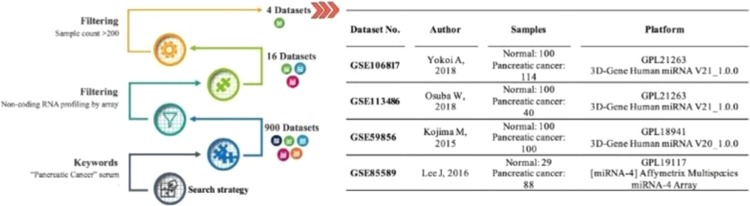
The selection procedure and information of the datasets. Note: Due to the increased number of datasets in the GEO database from the date of this investigation (February, 2019), different results may be obtained by applying the same criteria.

The flow chart of the datasets selection procedure and the features of the datasets are shown in Fig. 1. Some of the datasets contain a large set of expression profiles of various cancers, including PC. For these studies, a large number of healthy controls were included as normal controls, and we selected control samples based on the number of PC samples. GSE106817 consists of more than 2000 normal serum samples and 115 serum samples from PC patients (Yokoi et al., 2018). GSE113486 includes 200 normal and 40 PC serum samples (Usuba et al., 2019). GSE59856 includes 300 healthy controls and 100 serum samples from PC patients (Kojima et al., 2015). The fourth dataset, GSE85589 consisted 29 healthy subjects and 88 PC patients’ sera. It should be added that just the two later datasets included some available demographic features such as cancer stage, age, gender and CA19-9 levels of the patients, so it was not possible to evaluate the associations of the final DEMs to these kinds of features. Two other datasets (GSE112264 & GSE124158) consisting of serum miRNA profiles from PC patients and healthy controls (70 controls and 81 PC) were also considered as the validation set.

### Differentially expressed miRNAs (DEMs) and overlapped DEMs among 4 groups

**Figure 2:**
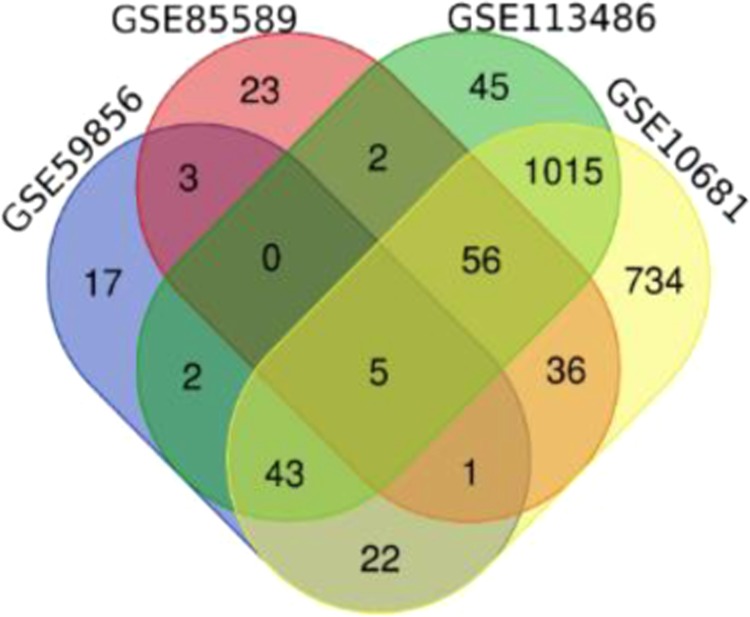
A VENN diagram representing the number of DEMs that are in common between different analyzed datasets.

A total of 1346, 1471, 127 and 93 miRNAs showed significant up/down regulation in GSE106817, GSE113486, GSE85589 and GSE59856 microarray datasets, respectively. After integration of the results, 105 miRNAs that were common in at least 3 of datasets were captured (Table 1). 5 miRNAs (hsa-let-7b-5p, hsa-miR-4721, hsa-miR-122-5p, hsa-miR-1290 and hsa-miR-125a-3p) were common between all four analyzed datasets. Figure 2 represents the number of miRNAs which are shared between the datasets.

**Table 1:** The overlapped DEMs among 4 different datasets.

GSE106817 GSE113486 GSE59856 GSE85589	GSE106817 GSE113486 GSE59856	GSE106817 GSE113486 GSE85589	GSE106817 GSE59856 GSE85589
hsa-let-7b-5p	hsa-miR-17-5p	hsa-miR-202-5p	hsa-miR-6075
hsa-miR-4721	hsa-miR-4732-5p	hsa-miR-4668-5p	
hsa-miR-122-5p	hsa-miR-575	hsa-miR-4708-3p	
hsa-miR-1290	hsa-miR-223-3p	hsa-miR-3171	
hsa-miR-125a-3p	hsa-miR-8073	hsa-miR-10a-3p	
	hsa-miR-6880-5p	hsa-miR-3910	
	hsa-miR-1246	hsa-miR-603	
	hsa-miR-6765-3p	hsa-miR-32-5p	
	hsa-miR-20b-5p	hsa-miR-532-5p	
	hsa-miR-20a-5p	hsa-miR-548u	
	hsa-miR-106a-5p	hsa-miR-4791	
	hsa-miR-6871-5p	hsa-miR-127-3p	
	hsa-miR-6893-5p	hsa-miR-6506-5p	
	hsa-miR-4454	hsa-miR-3927-3p	
	hsa-miR-451a	hsa-miR-4742-5p	
	hsa-miR-126-3p	hsa-miR-3128	
	hsa-miR-4476	hsa-miR-4696	
	hsa-let-7f-5p	hsa-miR-5195-3p	
	hsa-let-7d-5p	hsa-miR-548aq-3p	
	hsa-miR-1236-5p	hsa-miR-455-3p	
	hsa-miR-4530	hsa-miR-509-5p	
	hsa-miR-25-3p	hsa-miR-548ac	
	hsa-miR-564	hsa-miR-155-5p	
	hsa-miR-663a	hsa-miR-548×-3p	
	hsa-miR-3907	hsa-miR-130a-3p	
	hsa-miR-6872-3p	hsa-miR-22-3p	
	hsa-miR-6857-5p	hsa-miR-548aj-3p	
	hsa-miR-30b-3p	hsa-miR-542-3p	
	hsa-miR-7977	hsa-miR-7154-5p	
	hsa-miR-124-3p	hsa-miR-4797-5p	
	hsa-miR-134-3p	hsa-miR-890	
	hsa-miR-221-3p	hsa-miR-101-3p	
	hsa-miR-1469	hsa-miR-6840-5p	
	hsa-miR-7975	hsa-miR-642b-3p	
	hsa-miR-4648	hsa-miR-5100	
	hsa-miR-619-5p	hsa-miR-3201	
	hsa-miR-92a-2-5p	hsa-miR-606	
	hsa-miR-125b-1-3p	hsa-miR-570-3p	
	hsa-miR-26a-5p	hsa-miR-4757-5p	
	hsa-let-7a-5p	hsa-miR-5681a	
	hsa-let-7c-5p	hsa-miR-7852-3p	
	hsa-miR-4733-3p	hsa-miR-4536-3p	
	hsa-miR-16-5p	hsa-miR-4712-3p	
		hsa-miR-20b-3p	
		hsa-miR-4490	
		hsa-miR-450b-5p	
		hsa-miR-3152-3p	
		hsa-miR-548a-3p	
		hsa-miR-221-5p	
		hsa-miR-628-5p	
		hsa-miR-335-5p	
		hsa-miR-181a-5p	
		hsa-miR-130b-3p	
		hsa-miR-518d-3p	
		hsa-miR-1278	
		hsa-miR-4423-3p

### AUC analysis

**Figure 3:**
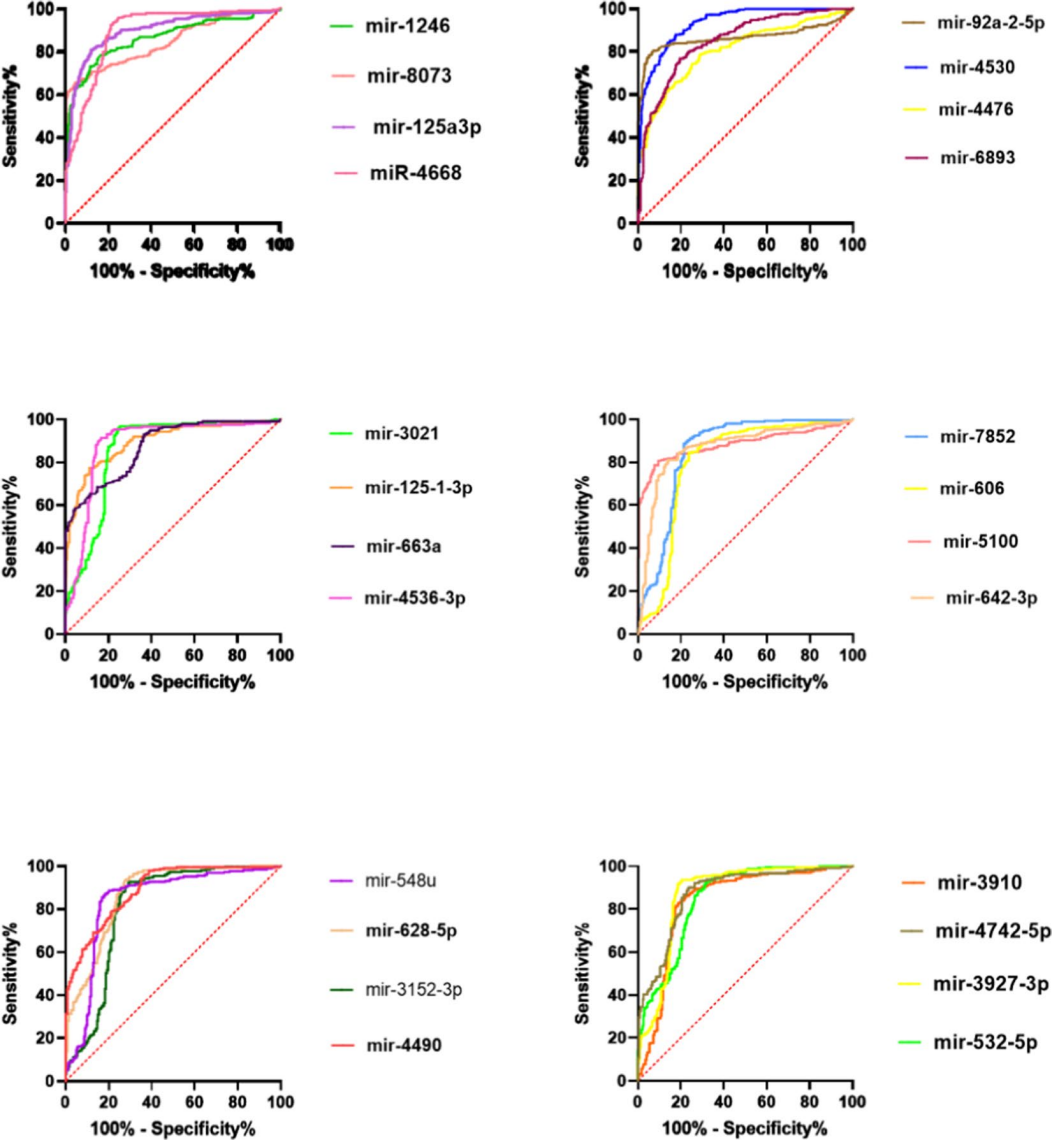
The ROC curves for DEMs with AUC > 80%.

After extraction of the expression values for all 105 considered DEMs, in order to find the most reliable ones in discriminating PC from healthy controls, AUC analysis was performed and the ROC curves for all 105 DEMs were prepared. Finally, 27 DEMs showed AUC score >80% (Table 2) and the ROC curves of them is represented in Fig. 3. The list of those DEMs with AUC >80% are also listed in Table 3. The most promising miRNA, miR-1469, was able to distinguish the two groups with 91% specificity and 100% sensitivity. ROC curve analysis showed that the AUC value for this miRNA was 0.98 (95% CI 0.95–1.06) and this value for miR-4530 was 0.93 (95% CI 0.91–0.97). Based on these results, miR-1469 and miR-4530 may be the strongest individual signatures for differentiating PC patients from healthy controls.

**Table 2:** The results of AUC analysis for considered DEMs.

Column1	Area	Std. Error	95% confidence interval	P value
hsa-miR-1469	**0.9825**	0.004891	0.9729 to 0.9921	<0.0001
hsa-miR-4530	**0.9332**	0.009712	0.9141 to 0.9522	<0.0001
hsa-miR-125a-3p	**0.901**	0.01227	0.8769 to 0.9250	<0.0001
hsa-miR-125-1-3p	**0.8952**	0.01372	0.8683 to 0.9221	<0.0001
hsa-miR-4668	**0.8904**	0.01581	0.8594 to 0.9214	<0.0001
hsa-miR-4490	**0.8903**	0.01431	0.8623 to 0.9184	<0.0001
hsa-miR-4536	**0.8863**	0.01757	0.8518 to 0.9207	<0.0001
hsa-miR-5100	**0.8833**	0.01674	0.8505 to 0.9161	<0.0001
hsa-miR-4742-5P	**0.8739**	0.01657	0.8414 to 0.9064	<0.0001
hsa-miR-663a	**0.8728**	0.01434	0.8447 to 0.9009	<0.0001
hsa-miR-628-5p	**0.8719**	0.01671	0.8392 to 0.9047	<0.0001
hsa-miR-3927-3P	**0.8709**	0.01821	0.8352 to 0.9066	<0.0001
hsa-miR-1246	**0.8694**	0.01583	0.8384 to 0.9004	<0.0001
hsa-miR-642-3p	**0.868**	0.01788	0.8330 to 0.9031	<0.0001
hsa-miR-92a-2-5p	**0.8618**	0.01883	0.8249 to 0.8987	<0.0001
hsa-miR-3021	**0.8549**	0.01928	0.8171 to 0.8927	<0.0001
hsa-miR-7852	**0.8536**	0.01927	0.8158 to 0.8914	<0.0001
hsa-miR-3128	**0.8521**	0.0195	0.8138 to 0.8903	<0.0001
hsa-miR-6893	**0.8503**	0.01614	0.8187 to 0.8819	<0.0001
hsa-miR-532-5p	**0.8499**	0.01802	0.8146 to 0.8852	<0.0001
hsa-mirR-8073	**0.8466**	0.01704	0.8132 to 0.8800	<0.0001
hsa-miR-548u	**0.8427**	0.02049	0.8026 to 0.8829	<0.0001
hsa-miR-3910	**0.8356**	0.02059	0.7953 to 0.8760	<0.0001
hsa-miR-4476	**0.8123**	0.0185	0.7760 to 0.8486	<0.0001
hsa-miR-4696	**0.8067**	0.02144	0.7647 to 0.8488	<0.0001
hsa-miR-3152	**0.8056**	0.02247	0.7616 to 0.8496	<0.0001
hsa-miR-606	**0.8022**	0.02274	0.7577 to 0.8468	<0.0001

**Table 3:** GO Enrichment of Target DEGs in PC.

GO Term	Total	Expected	Hits	Pval
response to drug	344	20.6	40	0.0363
regulation of translation	228	13.7	28	0.0427
cellular membrane organization	471	28.3	48	0.0427
cell-cell junction organization	186	11.2	24	0.0427
homeostasis of number of cells	196	11.8	25	0.0427
positive regulation of cellular component organization	560	33.6	54	0.0427
ER-nucleus signaling pathway	111	6.66	17	0.0427

### Co-expression correlations

**Figure 4:**
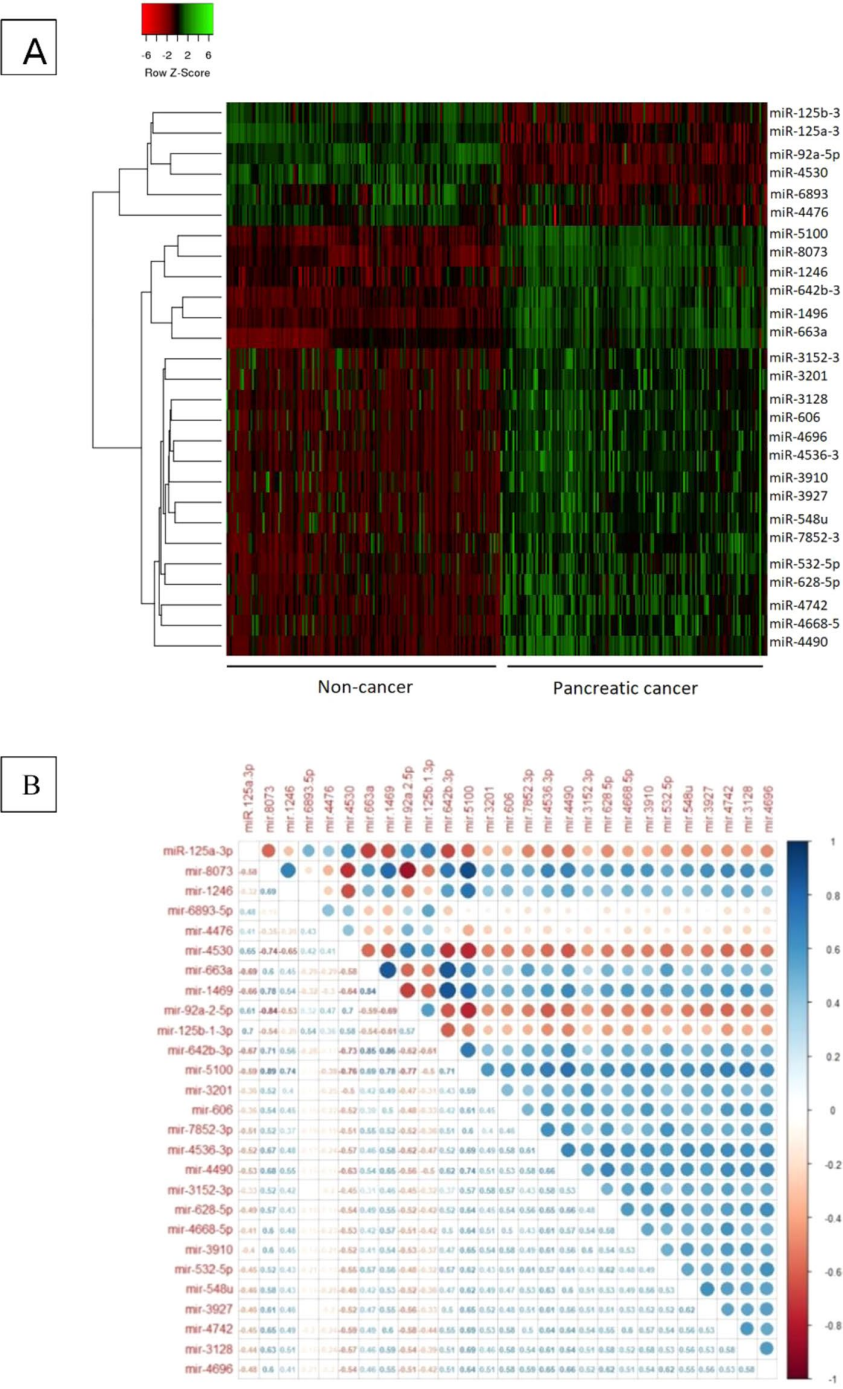
(**A**) The hierarchically clustered Heatmap built up using the expression values of all 27 captured DEMs. (**B**) Pearson’s correlation plot for 27 DEMs expression profiles. Pearson’s correlations calculated for all 27 DEMs values are demonstrated as circles whose sizes are representative of the certain correlation value, with colors ranging from dark red (coefficient -1), to dark blue (coefficient 1), as described in the color scale.

Figure 4A demonstrates a hierarchically clustered Heatmap built up using the expression values of all 27 captured DEMs. Six miRNAs showed down-regulation and 21 miRNA showed up-regulation in PC patients compared with healthy subjects. Pearson’s correlation coefficients were determined among all the 27 miRNA captured signatures (Fig. 4B). Altogether, 6 miRNAs including miR-5100, miR-8073, miR-642b-3p, miR-1246, miR-1469 and miR-663a showed the strongest positive correlations. The highest positive correlation coefficient was found between miR-5100 and miR-8073 (Pearson’s correlation = 0.893, p < 0.001), followed by miRNA-642b-3p with miR-663a and miR-1469 (Pearson’s correlations = 0.864, p < 0.001 and 0.851, p < 0.001).

### MiRNA-miRNA interaction network

**Figure 5:**
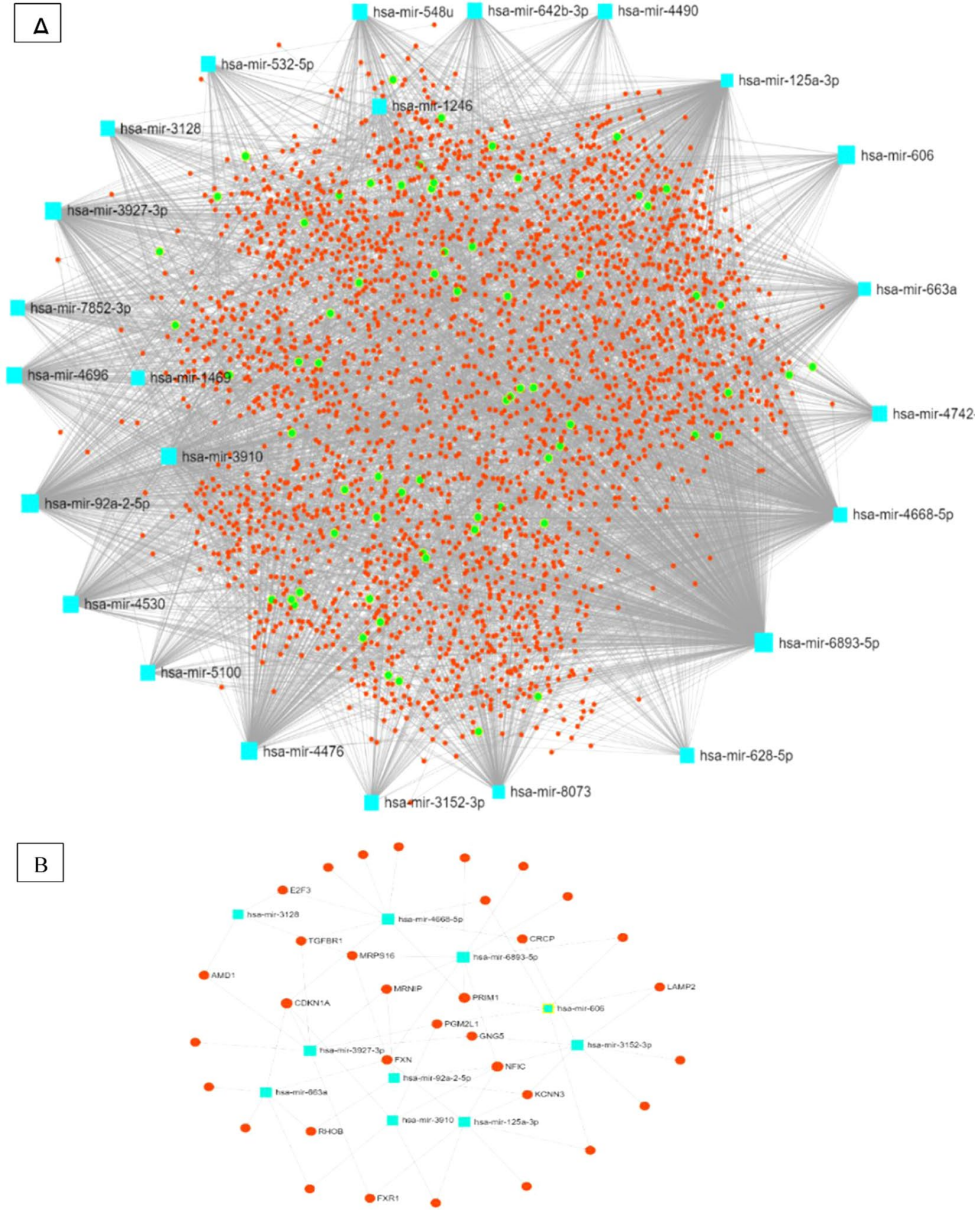
(**A**) The miRNA-mRNA interactions among all 27 considered DEMs. The red circles represent all predicted target genes and the green ones represent the genes implicated in cancer related pathways. (**B**) Well-known target genes for pancreatic cancer in associations to the considered DEMs.

Using MiRNet online software, the interactions between the 27 DEMs were analyzed based on their target genes and downstream molecular pathways. After uploading the miRNA IDs and setting the cut off degree on 2, a networks based on the following parameters was acquired (Fig. 5A): number of queries: 27, number of nodes: 2931 (miRNAs: 29, Targets: 2826) and number of edges:18640 (Fig. 5A). A module consisting of PC related genes and their connected miRNAs was extracted from this network and the result is represented in Fig. 5B.

### Target genes of miRNAs and functional enrichment analysis

Overall 1147 target genes for all 27 DEMs were predicted. The full list of those DEMs in addition to their predicted target genes are available in Supplementary File 1. Through DAVID online tool, GO and KEGG pathway enrichment of the identified target genes were performed. GO biological process (BP) analysis showed that the target genes were mostly involved in response to drug, regulation of translation and cellular membrane organization (Table 3). KEGG analysis showed that the target genes were mainly implicated in cancer-related pathways including chronic myeloid leukemia, glioma, prostate cancer and also pancreatic cancer (Table 4).

**Table 4:** KEGG pathway enrichment of Target DEGs in PC.

Pathway	Total	Expected	Hits	Pval
Chronic myeloid leukemia	73	4.59	19	1.28E-05
Glioma	65	4.08	17	2.01E-05
Prostate cancer	87	5.47	20	2.01E-05
Melanoma	68	4.27	17	3.05E-05
Colorectal cancer	49	3.08	13	0.000266
Non-small cell lung cancer	52	3.27	13	0.000452
Pathways in cancer	310	19.5	39	0.00048
Circadian rhythm - mammal	22	1.38	8	0.000881
Bladder cancer	29	1.82	9	0.00106
Pancreatic cancer	69	4.33	14	0.00158
HTLV-I infection	199	12.5	27	0.0019
p53 signaling pathway	68	4.27	13	0.00448
Endometrial cancer	44	2.76	10	0.00502
mTOR signaling pathway	45	2.83	10	0.00566
Acute myeloid leukemia	57	3.58	11	0.00985
Chagas disease (American trypanosomiasis)	89	5.59	14	0.0155
Small cell lung cancer	80	5.03	13	0.016
TGF-beta signaling pathway	84	5.28	13	0.024
Fc gamma R-mediated phagocytosis	97	6.09	14	0.0304
Jak-STAT signaling pathway	99	6.22	14	0.0351
Renal cell carcinoma	60	3.77	10	0.0386
Cell cycle	124	7.79	16	0.0428
Regulation of actin cytoskeleton	182	11.4	21	0.043

### PPI network construction and top module selection

**Figure 6:**
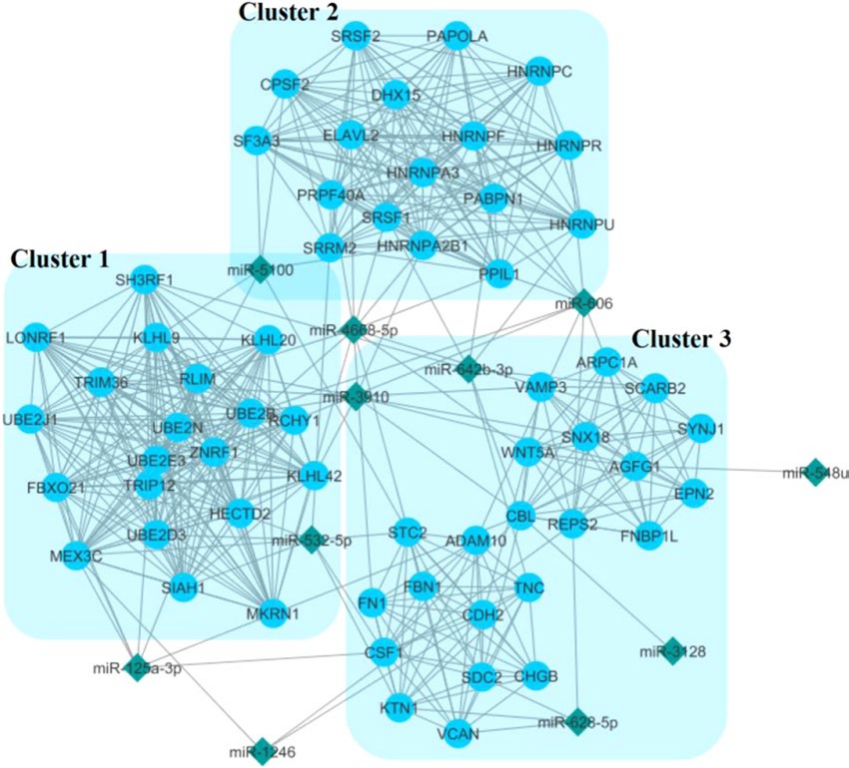
Three top modules (clusters) extracted from PPI network interactions between all of the target genes of considered miRNAs. Blue circles and the green diamonds represent the predicted target genes and the associated miRNAs respectively.

The PPI network was composed of 506 nodes and 10805 edges. Using the plug-in MCODE in Cytoscape software, three significant modules were selected with MCODE score ≥10 (Fig. 6). Functional enrichment analysis indicated that the genes of these modules were significantly enriched in ubiquitination, gene expression regulation, and spliceosome complexes.

### Top clustered DEMs and modules

Five clusters of most related miRNAs amongst all of the 27 considered DEMs were defined based on the different aspects of their trends in the expression and functional properties. The list of all clusters components is available in Supplementary File 2.

### Multivariate regression models

The results of univariable logistic regression showed the significant role of each miRNA in the distinction of PC (Supplementary File 2, Crude model). Univariable logistic regression analysis in each cluster showed that all selected miRNAs significantly increase the risk of PC and these associations were statistically highly significant (P < 0.0001; Supplementary File 2, Crude Model).

The multivariable logistic regression of miRNA expression was based on five cultures that resulted by clustering method in bioinformatics analysis. In each cluster when all predictors were included in the model, the association of them with PC changed. In cluster 1, while the strength of association among miR-125a-3p, miR-92a-2-5p and miR-4530 and PC decreased, but remained statistically significant (P = 0.031, P < 0.0001 and P < 0.0001, respectively; Supplementary Table 1, Model A). However, the association between miR-125b-1-3p, miR-6893-5p and miR-4476 and PC were statistically non-significant and omitted in further analysis. In cluster 2, the associations between miR-8073 and miR-663a and PC were decreased but remained statistically significant (P = 0.005 and P = 0.032, respectively; Supplementary Table 1, Model A). Although the interaction between miR-5100 and miR-642b-3p were found to be non-significant (P = 0.082 and P = 0.061, respectively; Supplementary Table 1, Model A), but they were forced into the final diagnostic model because of their trend relevance. However, the association between miR-1246 and PC was statistically non-significant and omitted from further analysis. In cluster 3, while the strength of association among PC and miR-125a-3p (P = 0.008; Supplementary Table 2, Model A) was increased and associations were decreased for miR-5100 and miR-642b-3p (P = 0.003 and P = 0.005, respectively; Supplementary Table 1, Model A), but remained statistically significant. However, the associations between miR-606, miR-4668 and miR-3910 and PC were statistically non-significant and omitted for further analysis. In cluster 4, the strength of association between PC and miR-4668, miR-663a and miR-125a-3p were increased (P = 0.078, P = 0.058 and P = 0.080 respectively; Supplementary Table 2, Model A), but remained statistically non-significant. Yet, they were forced into the final diagnostic model because of their trend relevance. However, associations between PC and other miRNAs of the cluster were statistically non-significant and omitted frm further analysis. In cluster 5, although the strength of association between miR-8073, miR-92a-25p and miR-5100 and PC was decreased (P = 0.004, P < 0.0001 and P = 0.006 respectively; Supplementary 2. Table 2, Model A), it remained statistically significant. However, association between PC and miR-1246 was statistically non-significant and omitted from further analysis.

The results of univariable and multivariable logistic regression along with the AIC and BIC values corresponding to the inclusion/exclusion of each predictor (Supplementary Table 2) were used to select predictors of the full diagnostic (logistic) model.

The optimum model in each cluster was selected by both methods corresponded to the model consisting of different predictors (Table 5). The final prediction models in different clusters leading to better diagnosis of PC are presented in Table 3. The Hosmer-Lemeshow statistic suggested that fit of model was adequate for each cluster dataset (Table 5). In this regard, the cluster 3 was the most fitted model that the ROC analysis on the predicted probabilities of PC derived from this model yielded an AUC of 0.95, Sensitivity 0.98 and Specificity 0.97, which segregated PC patients from controls. Our suggested model, based on miRNA expression indices provides a molecular screening strategy, suitable for application prior to subsequent invasive methods of risk monitoring, such as surgery. However, it should be noted that this paper is basically bioinformatics research. Without any new sample being prospectively collected and examined to prove the validity of the proposed signatures, the suggested models will not be appropriate for clinical use.

**Table 5:** Full diagnostic (logistic) model for pancreatic cancer, including the intercept.

Intercept and Predictors	Coefficient	SE	OR	95% CI	*P* value	VIF	Sensitivity	Specificity	Opt Cutoff
**Cluster 1**									
Intercept	20.04426	3.258	—	—					
mir125a3p	−0.103	0.029	0.90	0.85–0.95	<0.0001	1.88			
mir92a25p	−0.140	0.028	0.86	0.82–0.91	<0.0001	2.14			
mir4530	−0.111	0.028	0.89	0.84–0.94	<0.0001	2.34			
Mean VIF	2.12								
AIC/ BIC	63.79/ 79.28								
McFadden’s Pseudo R^2^	0.8853								
Hosmer-Lemeshow GOF	X^2^ = 1.30, *P* value= 0.9956								
ROC area (95% CI)	0.9964 (0.99287–0.99990)						0.98	0.95	0.576
**Cluster 2**									
Intercept	−22.92	5.751							
mir5100	0.080	0.045	1.08	0.99–1.18	0.079	5.85			
mir8073	0.152	0.052	1.16	1.05–1.29	0.004	5.47			
mir642b3p	0.107	0.056	1.11	0.99–1.24	0.056	4.64			
mir663a	0.126	0.053	1.13	1.02–1.26	0.019	4.15			
Mean VIF	5.03								
AIC/ BIC	31.13/ 50.50								
McFadden’s Pseudo R^2^	0.9565								
Hosmer- Lemeshow GOF	X^2^ = 11.81, *P* value= 0.1598								
ROC area (95% CI)	0.9995 (0.99844–1.00000)						0.99	0.98	0.553
**Cluster 3**									
Intercept	−3.793	3.393							
mir8073	0.22	0.051	1.25	1.13–1.38	<0.0001	1.26			
mir92a25p	0.17	0.037	1.19	1.10–1.28	<0.0001	2.02			
mir5100	−0.23	0.059	0.79	0.70–0.89	<0.0001	2.00			
Mean VIF	1.76								
AIC/ BIC	55.25611/ 70.74458								
McFadden’s Pseudo R2	0.9028								
Hosmer- Lemeshow GOF	X^2^ = 2.22, *P* value= 0.9735								
ROC area (95% CI)	0.9970 (0.99334–1.00000)						0.98	0.97	0.688
**Cluster 4**									
Intercept	−7.466	2.528							
mir4668	0.142	0.045	1.15	1.05–1.26	0.002	6.63			
mir663a	−0.11	0.027	0.89	0.84–0.94	<0.0001	3.51			
mir125a3p	0.109	0.039	1.11	1.03–1.20	0.005	4.65			
Mean VIF	4.93								
AIC/ BIC	49.65036/ 65.13883								
McFadden’s Pseudo R2	0.9144								
Hosmer- Lemeshow GOF	X^2^ = 2.22, *P* value= 0.9735								
ROC area (95% CI)	0.90 (0.89–0.95)						0.96	0.90	0.589
**Cluster 5**									
Intercept	3.966	5.754							
mir125a3p	−0.382	0.166	0.68	0.49–0.94	0.021	1.91			
mir5100	0.242	0.089	1.27	1.06–1.51	0.007	2.13			
mir642b3p	0.216	0.083	1.24	1.05–1.46	0.010	2.54			
Mean VIF	2.19								
AIC/ BIC	23.29147/ 38.77994								
McFadden’s Pseudo R2	0.9686								
Hosmer- Lemeshow GOF	X^2^ = 0.03, *P* value= 1.0000								
ROC area (95% CI)	0.95 (0.92–0.99)						0.97	0.93	0.401

**SE**: Standard error. **OR**: Odds Ratio. **X**^**2**^: Chi square statistic. **GOF**: Goodness of fit. **ROC** = Receiver-operating characteristic. **VIF**: variance inflation factor

### Validation

**Figure 7:**
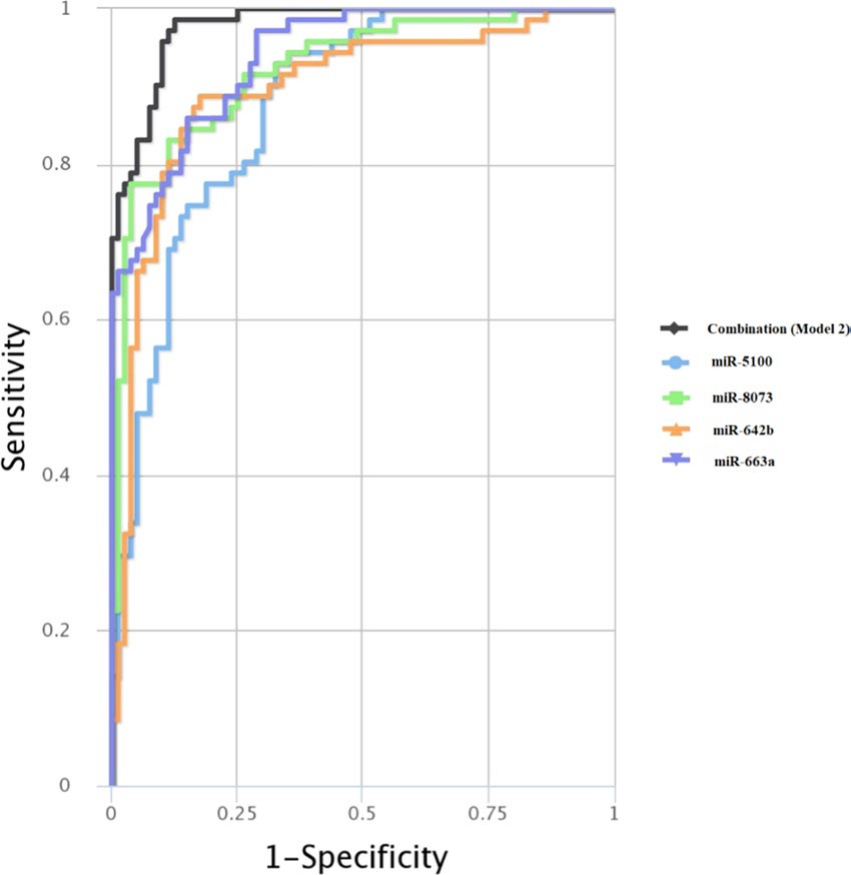
Performance of the model 2 on the validation cohort. The combinatorial multivariable ROC curve of the model is demonstrated. Colored lines represent the ROCs of each marker individually. Black line represents the combinatorial ROC for all of the DEMs as model 2.

The performance evaluations of the resulted diagnostic models showed that model 2 demonstrates the best performance in discriminating PC patients from healthy controls (AUC: 0.978; sensitivity: 0.986; specificity: 0.875) (Fig. 7 & Table 6). The 4 other models also showed AUC >80% which are statistically significant values (Table 6 & Supplementary File 3).

**Table 6:** The performance of the considered models on the validation set.

Symbol	AUC	SE	SP
Combination model 1	0.936	0.859	0.925
Combination model 2	0.978	0.986	0.875
Combination model 3	0.969	0.901	0.938
Combination model 4	0.957	0.831	0.938
Combination model 5	0.929	0.873	0.900

**AUC**: Area under curve, **SE**: Sensitivity, **SP**: Specificity.

## Discussion

**Figure 8:**
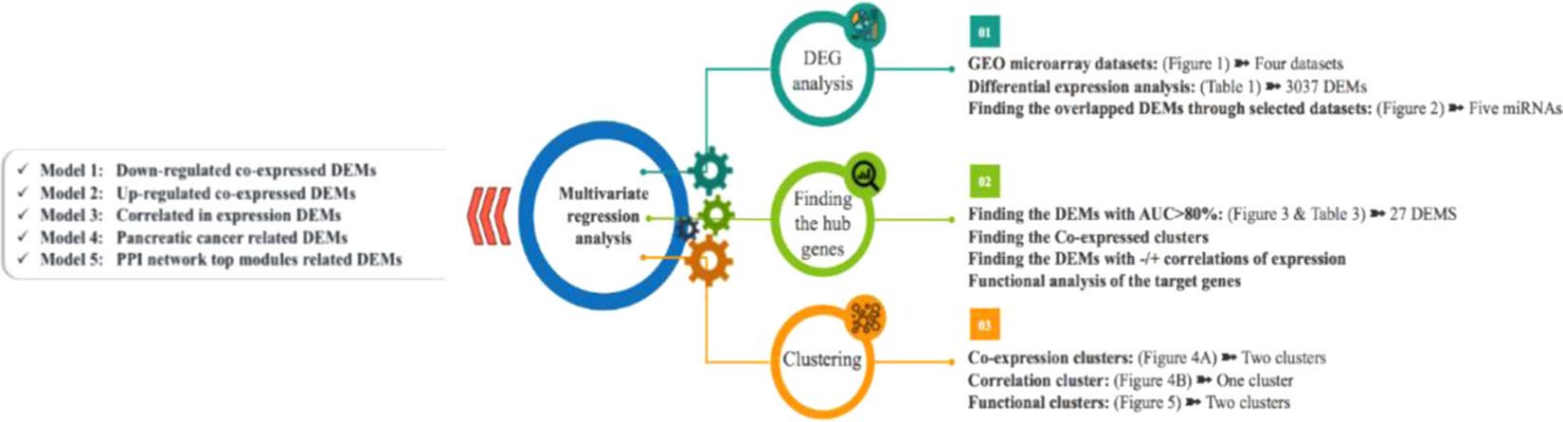
The summary of the whole steps of the hub DEMs selection and clustering of them.

In order to early diagnosis of patients with PC, there is a comprehensive need to find biomarkers with high specificity and sensitivity. By using bioinformatics methods, this study describes 5 new miRNA panels for PC diagnosis using a combination of 27 miRNAs in serum of PC patients. To find the miRNA candidates, 4 GEO microarray datasets containing the miRNA expression profiles from the serum of PC and healthy controls were chosen for differential expression analysis. DEMs from all of the datasets were extracted and at the result, 105 miRNAs that were common between 4 or 3 categories were captured out. In the next step, the expression values for all these DEMs were extracted and normalized. Using ROC curve analysis, the most powerful DEMs in discriminating PC patients from healthy controls were picked out. Overall, 27 DEMs showed AUC >80% and were considered as suitable candidates for further analysis. Although recent findings have introduced circulating miRNAs as diagnostic cancer markers, none have led to the utilization of these markers for clinical plans due to the insufficiency of individual miRNA biomarkers in clinical testing^22,23^. An increasing interest to combine biomarkers into unique panels tackles the problem of tumor heterogeneity and low specificity and sensitivity of single miRNAs to diagnose certain cancers. For this reason, multiple mathematical models have been employed to weigh the efficiency of combinations of miRNAs as cancer diagnostic biomarkers. These methods comprise threshold-based methods, logistic regression, decision trees and support vector machine^24^. In this study, we used logistic regression method to find the most promising miRNA combinations as diagnostic models for PC. Figure 8 demonstrates all steps of finding the hub DEMs and clustering processes.

To find the hub genes, multiple bioinformatics and statistical analyses were performed to specify the fittest miRNA combination among all 27 DEMs, as a diagnostic model. Five clusters of miRNAs were introduced based on different approaches in grouping genes such as clustering based on co-expression (cluster 1-2), correlations of the expressions (cluster 3), association of the target genes to PC (cluster 4) and functional enrichment analysis of the target genes (cluster 5). The first 2 clusters were determined based on the results of hierarchically clustered genes, where based on the co-expression of the DEMs, 2 clusters could be separated. The 3^th^ cluster is consisted of the genes that have the highest positive or negative correlation (>0.8) of the expression to each other. Based on the functional enrichment analyses that were performed on the predicted target genes of considered DEMs, two more clusters were specified. One of them included the DEMs that target well-known genes contributed to PC (cluster 4) and the second one consisted the DEMs that are associated with the top modules extracted from the PPI network of whole target genes of 27 DEMs (cluster 5). Finally, in order to find the most promising models amongst the determined clusters, multivariate cox regression analysis was performed on all clusters and the fittest models as panels of miRNAs were identified to discriminate PC patients from the healthy subjects. Overall, 2 models as the co-expression models, 1 model as the correlation model and 2 models as the gene functional models were introduced.

As can be seen in Fig. 4, two groups of co-expressed DEMs are prominent. The first cluster is down-regulated and the second one is up-regulated and both of them consist the DEMs with the most similarity in expression to each other. The downregulated co-expressed cluster consists of miR-125b-1-3p, miR-125a-3p, miR-92a-5p, miR-4530, miR-6893-5p and miR-4476. While all of these DEMs may be strong discriminators of PC patients from healthy controls solitarily, but the fittest model extracted from this cluster included miR-125a-3p, miR-92a-5p and miR-4530. These miRNAs have shown differential expression in a variety of cancers especially gastro Intestinal cancers in multiple studies and are known as tumor suppressor miRNAs^25–28^. The other DEMs of this cluster also have documented traces in association with GI cancers. For example, Yamada A et al. have introduced liquid biopsy markers for early detection of colorectal cancer and, in a cohort of 237 patients, circulating levels miR-125b independently showed differentiated expression in colorectal neoplasms in comparison to healthy controls. However, they showed that this miRNA in combination with some other miRNAs as a panel has improved the accuracy of detection^29^. The second cluster included a set of up-regulated miRNAs such as miR-1469, miR-1246, miR-5100, miR-8073, miR-642b-3p and miR-663a that showed significant co-expression in PC patients. MiR-1469 was the most powerful individual marker in the diagnosis of PC in the results of our analyses. Multiple studies have reported the effect and differential expression of this miRNA in various cancers such as lung, gastric, rectal and also pancreatic cancer^30–33^. Similarly, miR-1246 has been introduced as a significant serum/plasma marker in the diagnosis of a variety of cancers including esophageal squamous cell carcinoma, lung, prostate, colorectal and eventually pancreatic cancer^34–38^. MiR-5100 has been associated with risk of PC as 2 studies have reported differential expression of this miRNA in PC patients’ saliva and cell lines^39,40^. Despite that miR-8073 has been confirmed as a tumor suppressor miRNA in some cancers such as breast, ovarian and colorectal^41–43^, in this study we found significant up-regulation of this miRNA in the serum of PC patients. In line with the results of our study, mir-642b-3p have records of significant overexpression in the serum of PC patients in some recent studies^44–46^. For miR-663a, some studies have reported significant down-regulation in a variety of cancers such as colorectal and non-small cell lung as well as PC^33,47,48^ that may show a tumor suppressive effect of this gene, but in this study we detected significant up-regulation of this miRNA through the analyzed datasets. Stablished model from cluster 2, consisted miR-5100, miR-8073, miR-642b-3p and miR-663a. Although miR-1469 and miR-1246 individually showed high power to differentiate PC patients from healthy controls, they did not fit into the logistic model along with other miRNAs. The correlation model was also the top model introduced from the third cluster including the DEMs with the strongest negative or positive correlation of expression. MiR-125a-3p, miR-606, miR-4668, miR-3910, miR-5100, miR-642b-3p and miR-532 formed this cluster and most of them have been reported as feasible diagnostic biomarkers for a variety cancers (Bibi et al., 2016b; Cheng, Wang, Han, & sciences, 2017; Song, Wang, Jin, Wang, & Duan, 2015), but here we introduced them as a unified model for this aim. The acquired logistic model from this cluster includes miR-125a-3p, miR-5100, miR-642b-3p that were discussed earlier.

In order to find miRNAs that are the most related to each other and can be used as a diagnostic panel, we performed multiple functional enrichment analyses so we could categorize the considered miRNAs based on the functions of their target genes. First, using an online miRNA target gene prediction software, all feasible target genes were predicted. Afterwards, the functional status of all target gene were analyzed in regard of GO and KEGG functional enrichment. GO analysis showed that a significant number of all target genes have role in the processes such as cellular membrane organization, cell-cell junction organization and. These kinds of cellular and molecular functions are clearly understood as critical procedures in tumorigenesis and metastasis^49,50^. On the other hand, the results of KEGG pathway analysis demonstrated that most of the target genes of considered miRNAs are implicated in various cancer-related pathways including chronic myeloid leukemia, glioma, prostate cancer and also PC. Figure 5A represents all miRNA-target genes interactions while the green circles represents the target genes implicated in cancer related pathways. Figure 5B is the extracted module consists of known genes correlated to PC as well as their contributed miRNAs. MiRNAs of this module were considered as a cluster and miR-4668, miR-663a, miR-3128, miR-125a-3p, miR-3910, miR-3152, miR-606, miR-3927 and miR-6893-5p were the compartments of it. After performing multivariate logistic regression analysis miR-4668, miR-663a and miR-125a-3p were unified as the fifth model. Amongst the 3 compartments of this model, miR-4668 has been shown to have over-expression in serum and tissue samples from in hepatocellular carcinoma and gastric cancer^51,52^. As it is shown in Fig. 5B, a group of considered miRNAs in this study may target some known genes associated with pancreatic cancer. For example, Schutte et al. detected improper hypermethylation of the p16/*CDKN1A* gene in a group of PC patients^53^. In this study, we found significant overexpression of miR-4668 and miR-663a that both are strongly predicted to target the *CDKN1A* gene. Another gene of this module is *RHOB*, that is a known tumor suppressor gene in various cancers^54,55^, nevertheless, it is not much known in PC. The revival of suppressed RHOB leads to tumor regression in different types of cancers^56^ and may be used as a critical target in cancer therapy^57^. Yonggang Tan et al. demonstrated significant down-regulation of RHOB in human PC and showed that this gene suppresses the progression of PC by inhibiting proliferation, migration, and invasion, as well as by inducing apoptosis^58^. In the present study, we showed that miR-92a-5p and miR-663a are predicted to target this gene and may have associations to PC progression.

In the aspect of PPI network construction through the target genes of considered DEMs and analysis of the top modules, we identified 3 top modules that had the strongest PPIs through all the target genes. Later, we found that a set of miRNAs are connected to all these top 3 modules, as it is shown in Fig. 8 (miR-8073, miR-92a-2-5p, miR-5100, miR-1246, miR-1469 and miR-642b-3p), so we assumed them as a cluster and the fifth model consisting of miR-8073, miR-92a-5p and miR-5100 were extracted from this cluster. The functional enrichment analyses showed that the most component of this three modules are implicated in ubiquitination, gene expression regulation and spliceosome complexes.

In conclusion, this study supports the accuracy of some formerly proposed biomarkers for PC and also has suggested new candidate miRNAs which can be used as diagnostic or prognostic means or as therapeutic targets. We introduced 5 diagnostic models consisting of different combinations of miRNAs, based on their significant expression algorithms and functional properties. The aim of this study was to identify appropriate miRNA biomarkers in serum samples that could differentiate PC from healthy individuals. For this matter, we have to first test the sides of the coin (ie, PC vs healthy controls) because if panels of microRNA could not discriminate these two extremes, it would not be possible to develop a diagnostic microRNA for early detection of primary tumors or early stages of the disease. However, it should be considered that none of these models have been tested in experimental studies up to now and they need to be validated in such investigations. Even though this bioinformatics study presented some additional biomarkers or panels for possible consideration in future research, the analyses in these datasets do not support the immediate clinical use of these biomarkers without more rigorous testing in large case-control and cohort studies. Besides, in order to reach to compatible results, researchers should avoid contaminations. As miRNAs can be found in the serum in different forms such as free, associated to HDL or enclosed in exosomes or micro vesicles, researchers should be careful in the isolation step of miRNAs from the desired samples.

## Supplementary information

Supplementary information 1.

Supplementary information 2.

Supplementary information 3.

Supplementary information 4.
