# Supplementary material for: Identification of potential microRNA panels for pancreatic cancer diagnosis using microarray datasets and bioinformatics methods
Source: Sci Rep. 2020 May 5;10:7559. doi: 10.1038/s41598-020-64569-1 (PMC7200710; doi:10.1038/s41598-020-64569-1)
Supplement: Supplementary file 2 — Supplementary information 2. [file 41598_2020_64569_MOESM2_ESM.docx]

**Multivariate regression analysis results**

**Identification of potential microRNA panels for pancreatic cancer diagnosis using microarray datasets and bioinformatics methods**

Roshanak Shams^1,2*^, Samaneh Saberi^1,3^, Amir Sadeghi^1^, Mohammadreza Zali^1^, Soudeh Ghafouri-Fard^2^, Hamid Asadzadeh Aghdaei^1*^

1-Research Center of Gastroenterology and Liver Disease, Shahid Beheshti University of Medical Sciences, Tehran, Iran

2-Department of Medical Genetics, Shahid Beheshti University of Medical Sciences,Tehran, Iran

3. HPGC Research Group, Medical Biotechnology Department, Biotechnology Research Center, Pasteur Institute of Iran, Tehran, Iran

Supplementary 2. Table 1**: Association between each predictor (miRNA) and Pancreatic cancer**

| **Characteristic** | **Crude model** | | **Model A*** | |  |
| --- | --- | --- | --- | --- | --- |
|  | **OR (95% CI)** | ***P* value** | **OR (95% CI)** | ***P* value** | **VIF** |
| **Cluster 1** |  |  |  |  |  |
| **mir125b13p** | 0.9264582 (0.912664- 0.9404609) | <0.0001 | 0.9871879 (0.9527392- 1.022882) | 0.477 | 2.35 |
| **mir125a3p** | 0.8431176 (0.8122333- 0.8751762) | <0.0001 | 0.9329033 (0.8757599- 0.9937754) | 0.031^⯎^ | 2.50 |
| **mir92a25p** | 0.8043391 (0.7675183- 0.8429264) | <0.0001 | 0.8619117 (0.8097756- 0.9174045) | <0.0001^⯎^ | 2.40 |
| **mir4530** | 0.8315114 (0.7996298- 0.8646641) | <0.0001 | 0.8956104 (0.8456653- 0.9485054) | <0.0001^⯎^ | 2.42 |
| **mir68935p** | 0.9505416 (0.9376741- 0.9635858) | <0.0001 | 0.9791643 (0.9332407- 1.027348) | 0.390 | 1.62 |
| **mir4476** | 0.8797166 (0.851698- 0.9086568) | <0.0001 | 0.9536685 (0.8609136- 1.056417) | 0.364 | 1.46 |
| **Cluster 2** |  |  |  |  |  |
| **mir5100** | 1.215851 (1.160155- 1.274221) | <0.0001 | 1.084671 (0.9898547- 1.18857) | 0.082^⯎^ | 7.20 |
| **mir8073** | 1.298621 (1.207904- 1.396151) | <0.0001 | 1.162553 (1.045958- 1.292145) | 0.005^⯎^ | 6.19 |
| **mir1246** | 1.102418 (1.080353- 1.124933) | <0.0001 | 1.006099 (0.9325813- 1.085412) | 0.875 | 2.42 |
| **mir642b3p** | 1.284376 (1.208566- 1.364941) | <0.0001 | 1.112494 (0.9952937- 1.243496) | 0.061^⯎^ | 5.32 |
| **mir663a** | 1.209197 (1.155352- 1.265551) | <0.0001 | 1.139717 (1.011176- 1.284597) | 0.032^⯎^ | 5.35 |
| **mir1469** | 1.367001 (1.246799- 1.498792) | <0.0001 | Not included in the model | - | 6.36 |
| **Cluster 3** |  |  |  |  |  |
| **mir8073** | 1.298621 (1.207904- 1.396151) | <0.0001 | 1.146106 (1.045182- 1.256776) | 0.004^⯎^ | 7.24 |
| **mir92a25p** | 0.8043391 (0.767518- 0.8429264) | <0.0001 | 0.8830759 (0.8248044- 0.9454642) | <0.0001^⯎^ | 3.65 |
| **mir5100** | 1.215851 (1.160155- 1.274221) | <0.0001 | 1.115982 (1.032774- 1.205895) | 0.006^⯎^ | 6.28 |
| **mir1246** | 1.102418 (1.080353- 1.124933) | <0.0001 | 1.019923 (.9494032- 1.095681) | 0.589 | 2.44 |
| **mir1469** | 1.367001 (1.246799- 1.498792) | <0.0001 | Not included in the model | - | 5.21 |
| **mir642b3p** | 1.284376 (1.208566- 1.364941) | <0.0001 | Not included in the model | - | 3.94 |
| **Cluster 4** |  |  |  |  |  |
| **mir4668** | 1.08601 (1.067104- 1.105251) | <0.0001 | 1.231846 (0.9765899- 1.553821) | 0.078^⯎^ | 1.81 |
| **mir663a** | 1.209197 (1.155352- 1.265551) | <0.0001 | 1.254398 (0.992508- 1.585392) | 0.058^⯎^ | 2.14 |
| **mir3128** | 1.112578 (1.088884- 1.136788) | <0.0001 | 1.102964 (0.9082489- 1.339422) | 0.323 | 2.00 |
| **mir125a3p** | 0.8431176 (0.8122333- 0.8751762) | <0.0001 | 0.7672365 (0.57043- 1.031944) | 0.080^⯎^ | 2.51 |
| **mir3910** | 1.095161 (1.07435- 1.116376) | <0.0001 | 1.157135 (0.8634836- 1.550651) | 0.328 | 2.07 |
| **mir3152** | 1.059882 (1.045522- 1.074439) | <0.0001 | 1.019912 (0.8773162- 1.185686) | 0.797 | 1.97 |
| **mir606** | 1.081393 (1.06282- 1.100291) | <0.0001 | 1.13972 (0.9297348- 1.397132) | 0.208 | 1.93 |
| **mir68935p** | 0.9505416 (0.9376741- 0.9635858) | <0.0001 | 0.8440129 (0.6819256- 1.044627) | 0.119 | 1.35 |
| **mir3927** | 1.129216 (1.101195- 1.15795) | <0.0001 | 1.158231 (0.910881- 1.47275) | 0.231 | 1.87 |
| **Cluster 5** |  |  |  |  |  |
| **mir125a3p** | 0.9264582 (0.912664- 0.9404609) | <0.0001 | 0.8866896 (0.8108053- 0.9696761) | 0.008^⯎^ | 1.92 |
| **mir606** | 1.081393 (1.06282- 1.100291) | <0.0001 | 1.061528 (0.9627684- 1.170418) | 0.231 | 1.85 |
| **mir4668** | 1.08601 (1.067104- 1.105251) | <0.0001 | 1.007862 (0.9358916- 1.085367) | 0.836 | 1.80 |
| **mir3910** | 1.095161 (1.07435- 1.116376) | <0.0001 | 1.00354 (0.9022028- 1.116261) | 0.948 | 1.95 |
| **mir5100** | 1.215851 (1.160155- 1.274221) | <0.0001 | 1.208145 (1.065071- 1.370438) | 0.003^⯎^ | 3.49 |
| **mir642b3p** | 1.284376 (1.208566- 1.364941) | <0.0001 | 1.200237 (1.056471- 1.363567) | 0.005^⯎^ | 2.65 |
| **mir532** | 1.08954 (1.071003- 1.108397) | <0.0001 | Not included in the model | - | 1.82 |
| *Adjusted for all variables within the cluster, ⯎Selected predictors for the final model (P≤0.1), VIF: variance inflation factor | | | | | |

Supplementary 2. Table 2: **Fitting Criterion values for the stepwise inclusion of each candidate predictor (miRNA)**

|  | **Candidate predictors** | **Stepwise Inclusion** | |  |  |  |  |  |
| --- | --- | --- | --- | --- | --- | --- | --- | --- |
|  | **Model** | **AIC** | **BIC** | **McFadden's Pseudo R2** | **LR chi2** | **P value** | **Log likelihood** | **Hosmer-Lemeshow GOF, P value** |
| **Cluster 1** | **mir125b13p** | 299.9898 | 307.734 | 0.3915 | 190.43 | <0.0001 | -147.9949 | 2.73, 0.9499 |
|  | **mir125b13p mir125a3p** | 197.4248 | 209.0411 | 0.6065 | 294.99 | <0.0001 | -95.712391 | 6.62, 0.5777 |
|  | **mir125b13p mir125a3p mir92a25p** | 85.22991 | 100.7184 | 0.8412 | 409.18 | <0.0001 | -38.614956 | 23.47, 0.0028 |
|  | **mir125b13p mir125a3p mir92a25p mir4530** | 64.75302 | 84.11361 | 0.8874 | 431.66 | <0.0001 | -27.376511 | 1.24, 0.9963 |
|  | **mir125b13p mir125a3p mir92a25p mir4530** **mir68935p** | 64.64321 | 87.87592 | 0.8918 | 433.77 | <0.0001 | -26.321607 | 12.38, 0.1352 |
|  | **mir125b13p mir125a3p mir92a25p mir4530 mir68935p mir4476** | 65.78736 | 92.89219 | 0.8935 | 434.63 | <0.0001 | -25.893682 | 15.20, 0.0554 |
|  |  |  |  |  |  |  |  |  |
| **Cluster 2** | **mir5100** | 114.6145 | 122.3587 | 0.7726 | 375.80 | <0.0001 | -55.30724 | 4.71, 0.7878 |
|  | **mir5100 mir8073** | 70.29845 | 81.9148 | 0.8678 | 422.12 | <0.0001 | -32.149223 | 11.87, 0.1570 |
|  | **mir5100 mir8073 mir1246** | 69.64079 | 85.12926 | 0.8733 | 424.77 | <0.0001 | -30.820393 | 57.69, <0.0001 |
|  | **mir5100 mir8073 mir1246 mir642b3p** | 38.46171 | 57.8223 | 0.9415 | 457.95 | <0.0001 | -14.230855 | 4.56, 0.8030 |
|  | **mir5100 mir8073 mir1246 mir642b3p mir663a** | 33.11486 | 56.34756 | 0.9566 | 465.30 | <0.0001 | -10.557429 | 12.70, 0.1225 |
|  | **mir5100 mir8073 mir1246 mir642b3p mir663a mir1469** | - | - | - | - | - | - | - |
|  |  |  |  |  |  |  |  |  |
| **Cluster 3** | **mir8073** | 76.02333 | 83.76756 | 0.8519 | 414.39 | <0.0001 | -36.011663 | 12.41, 0.1336 |
|  | **mir8073 mir92a25p** | 57.07408 | 68.69043 | 0.8950 | 435.34 | <0.0001 | -25.537039 | 1.88, 0.9845 |
|  | **mir8073 mir92a25p mir5100** | 49.65036 | 65.13883 | 0.9144 | 444.76 | <0.0001 | -20.82518 | 2.22, 0.9735 |
|  | **mir8073 mir92a25p mir5100 mir1246** | 51.35004 | 70.71063 | 0.9150 | 445.06 | <0.0001 | -20.675019 | 2.92, 0.9395 |
|  | **mir8073 mir92a25p mir5100 mir1246 mir642b3p** | - | - | - | - | - | - | -- |
|  | **mir8073 mir92a25p mir5100 mir1246 mir642b3p mir1469** | - | - | - | - | - | - | -- |
|  |  |  |  |  |  |  |  |  |
|  |  |  |  |  |  |  |  |  |
| **Cluster 4** | **mir4668** | 336.737 | 344.4813 | 0.3159 | 153.68 | <0.0001 | -166.36852 | 105.77, <0.0001 |
|  | **mir4668 mir663a** | 99.3145 | 110.9308 | 0.8082 | 393.10 | <0.0001 | -46.657248 | 3.11, 0.9274 |
|  | **mir4668 mir663a mir3128** | 78.94401 | 94.43248 | 0.8541 | 415.47 | <0.0001 | -35.472005 | 1.30, 0.9955 |
|  | **mir4668 mir663a mir3128 mir125a30** | 40.63106 | 59.99165 | 0.9370 | 455.78 | <0.0001 | -15.315528 | 0.36, 1.0000 |
|  | **mir4668 mir663a mir3128 mir125a30 mir3910** | 37.6485 | 60.88121 | 0.9473 | 460.77 | <0.0001 | -12.82425 | 0.60, 0.9997 |
|  | **mir4668 mir663a mir3128 mir125a30 mir3910 mir3152** | 38.47642 | 65.58124 | 0.9497 | 461.94 | <0.0001 | -12.23821 | 0.61, 0.9997 |
|  | **mir4668 mir663a mir3128 mir125a30 mir3910 mir3152 mir606** | 38.02293 | 68.99988 | 0.9547 | 464.39 | <0.0001 | -11.011466 | 0.40, 0.9999 |
|  | **mir4668 mir663a mir3128 mir125a30 mir3910 mir3152 mir606 mir68935p** | 36.16741 | 71.01647 | 0.9627 | 468.25 | <0.0001 | -9.0837033 | 0.17, 1.0000 |
|  | **mir4668 mir663a mir3128 mir125a30 mir3910 mir3152 mir606 mir68935p mir3927** | 34.89775 | 73.61893 | 0.9694 | 471.52 | <0.0001 | -7.4488762 | 0.10, 1.0000 |
|  |  |  |  |  |  |  |  |  |
| **Cluster 5** | **mir125a3p** | 207.1642 | 214.9085 | 0.5823 | 283.25 | <0.0001 | -101.58211 | 2.73, 0.9499 |
|  | **mir125a3p mir606** | 132.6556 | 144.272 | 0.7396 | 359.76 | <0.0001 | -63.327811 | 4.62, 0.7974 |
|  | **mir125a3p mir606 mir4668** | 85.64066 | 101.1291 | 0.8404 | 408.77 | <0.0001 | -38.820329 | 4.90, 0.7680 |
|  | **mir125a3p mir606 mir4668 mir3910** | 60.27986 | 79.64045 | 0.8966 | 436.14 | <0.0001 | -25.13993 | 0.91, 0.9988 |
|  | **mir125a3p mir606 mir4668 mir3910 mir5100** | 31.17456 | 54.40727 | 0.9606 | 467.24 | <0.0001 | -9.5872801 | 0.08, 1.0000 |
|  | **mir125a3p mir606 mir4668 mir3910 mir5100 mir642b3p** | 21.21068 | 48.3155 | 0.9852 | 479.20 | <0.0001 | -3.6053385 | 0.00, 1.0000 |
|  | **mir125a3p mir606 mir4668 mir3910 mir5100 mir642b3p mir532** | - | - | - | - | - | - | - |

**AIC:** Akaike Information Criterion; **BIC:** Bayesian Information Criterion; **GOF**: Goodness of fit
