# Supplementary material for: Identification of potential microRNA panels for pancreatic cancer diagnosis using microarray datasets and bioinformatics methods
Source: Sci Rep. 2020 May 5;10:7559. doi: 10.1038/s41598-020-64569-1 (PMC7200710; doi:10.1038/s41598-020-64569-1)

**Performance analysis of the considered models on the validation set**

**Identification of potential microRNA panels for pancreatic cancer diagnosis using microarray datasets and bioinformatics methods**

Roshanak Shams^1,2*^, Samaneh Saberi^1,3^, Amir Sadeghi^1^, Mohammadreza Zali^1^, Soudeh Ghafouri-Fard^2^, Hamid Asadzadeh Aghdaei^1*^

1-Research Center of Gastroenterology and Liver Disease, Shahid Beheshti University of Medical Sciences, Tehran, Iran

2-Department of Medical Genetics, Shahid Beheshti University of Medical Sciences,Tehran, Iran

3. HPGC Research Group, Medical Biotechnology Department, Biotechnology Research Center, Pasteur Institute of Iran, Tehran, Iran

**Model 1**


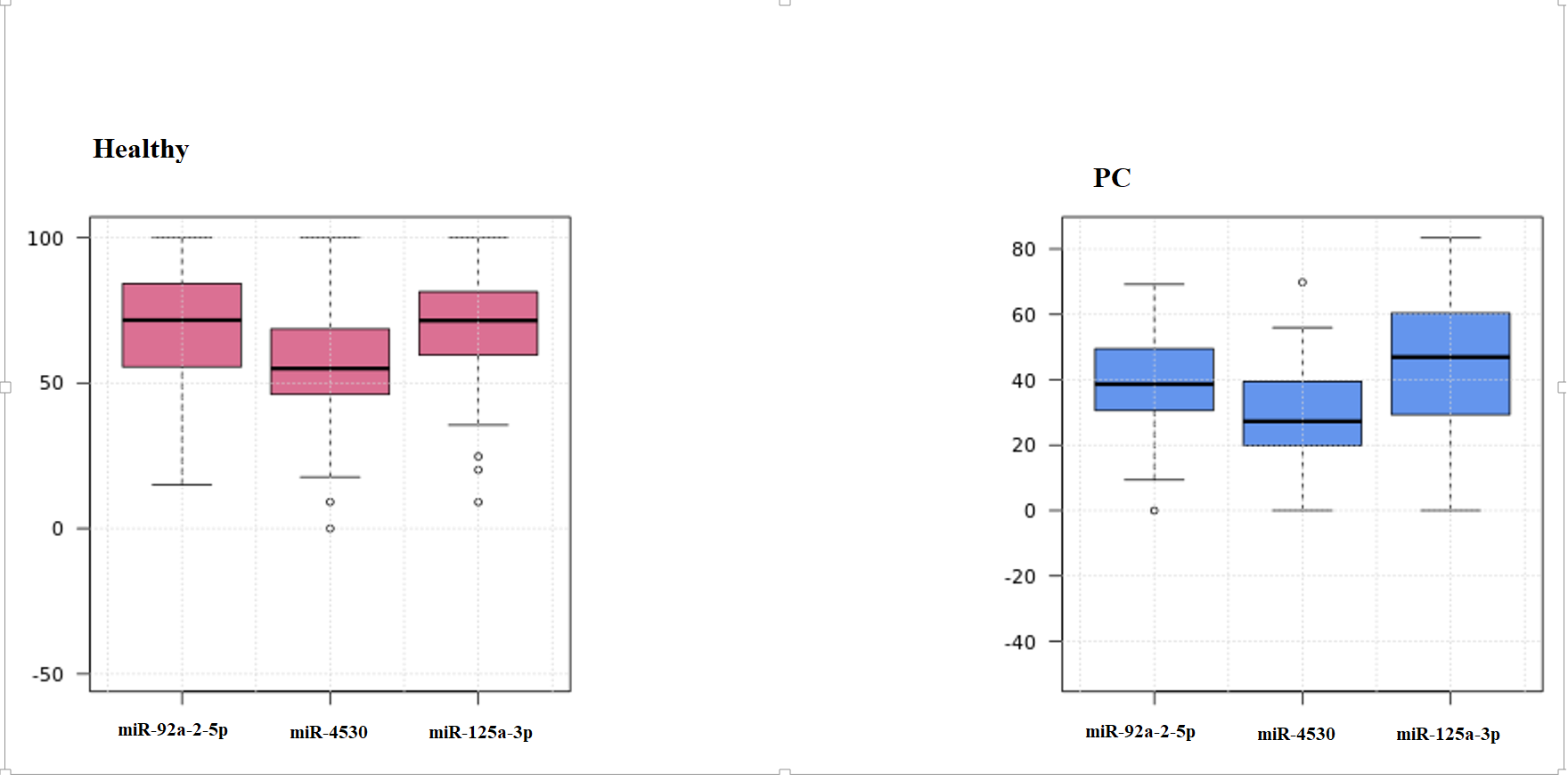


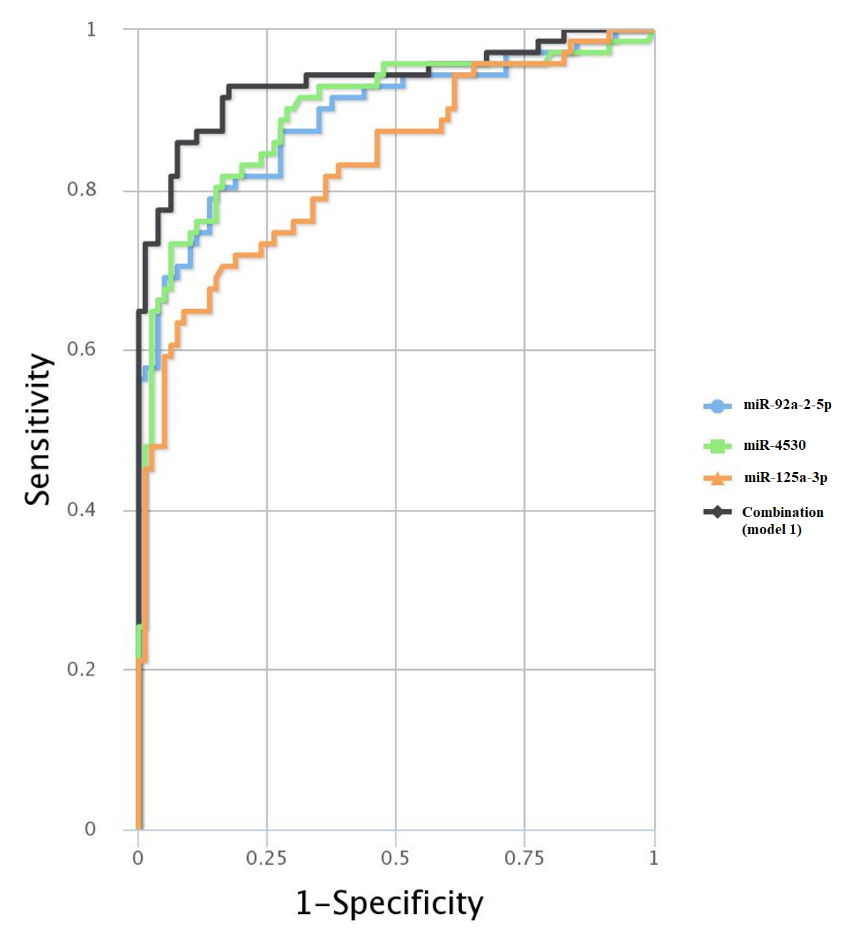


| **Symbol** | **AUC** | **SE** | **SP** | **Opt Cutoff** |
| --- | --- | --- | --- | --- |
| miR-92a-2-5p | 0.890 | 0.803 | 0.850 | 0.537 |
| miR-4530 | 0.894 | 0.732 | 0.938 | 0.617 |
| miR-125a-3p | 0.832 | 0.648 | 0.912 | 0.613 |
| Combo IV | 0.936 | 0.859 | 0.925 | 0.509 |

**
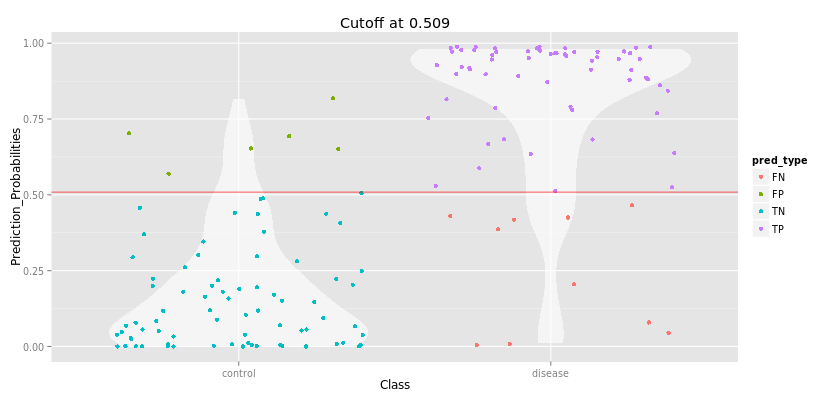
**

////////////////////////////////////////////////////////////////////////////////////////////////////////////////////////

Model 2


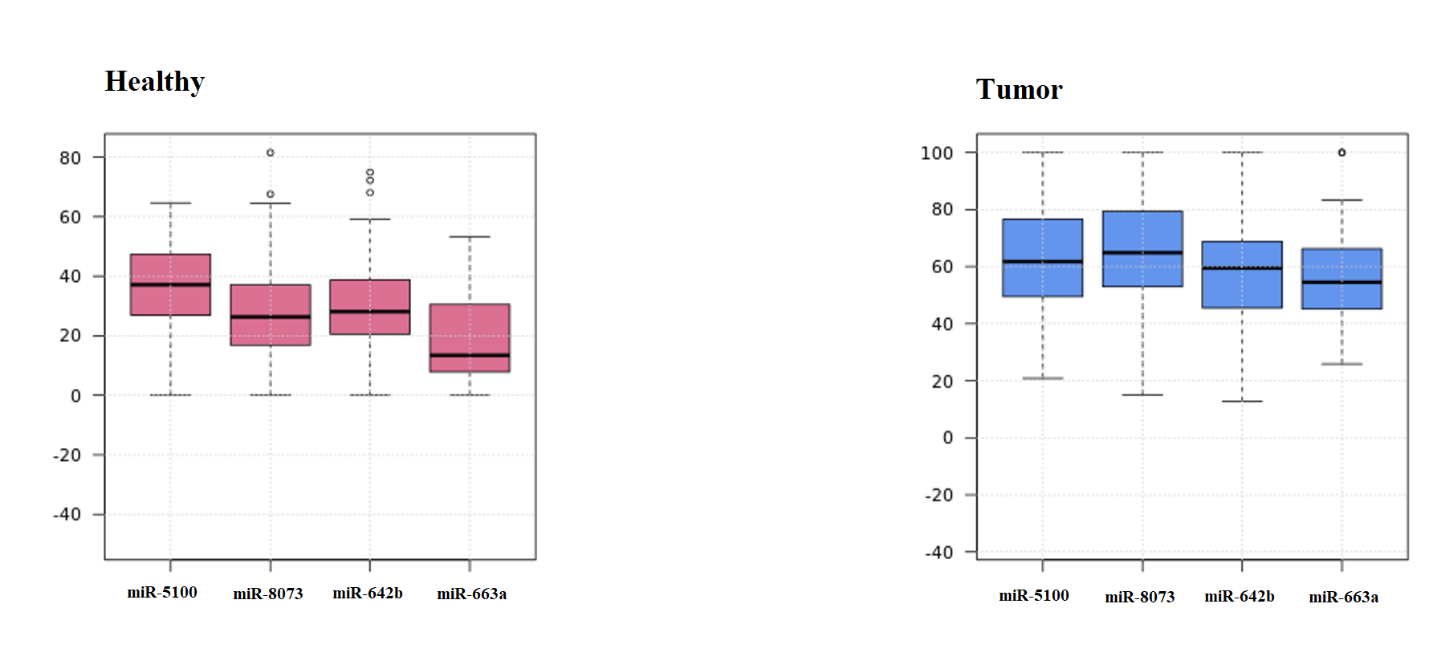


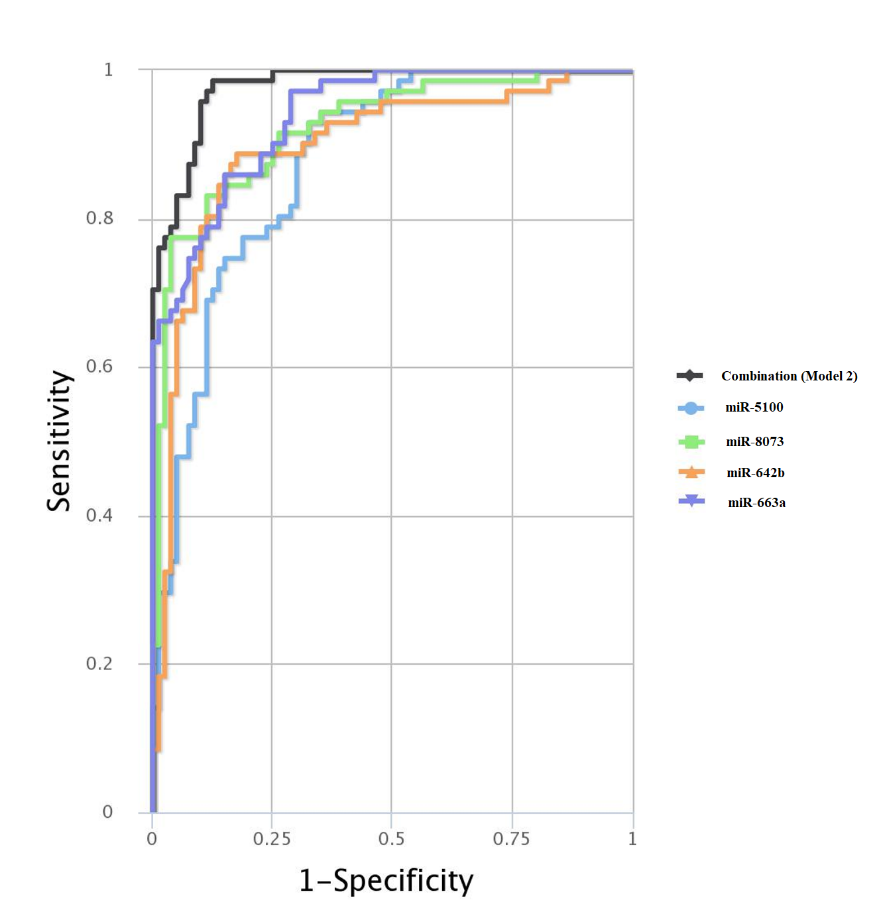


Multivariable ROC cureves

Scores

| **Symbol** | **AUC** | **SE** | **SP** | **Opt Cutoff** |
| --- | --- | --- | --- | --- |
| miR-5100 | 0.877 | 0.930 | 0.675 | 0.307 |
| miR-8073 | 0.923 | 0.775 | 0.962 | 0.644 |
| miR-642b | 0.895 | 0.887 | 0.825 | 0.403 |
| miR-663a | 0.939 | 0.859 | 0.850 | 0.354 |
| Combo model 2 | 0.978 | 0.986 | 0.875 | 0.207 |

Predictions


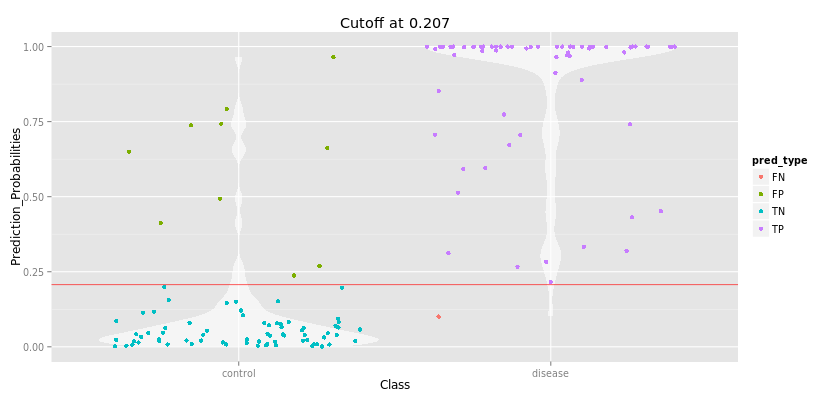


////////////////////////////////////////////////////////////////////////////////////////////////////////////////////////

Model 3


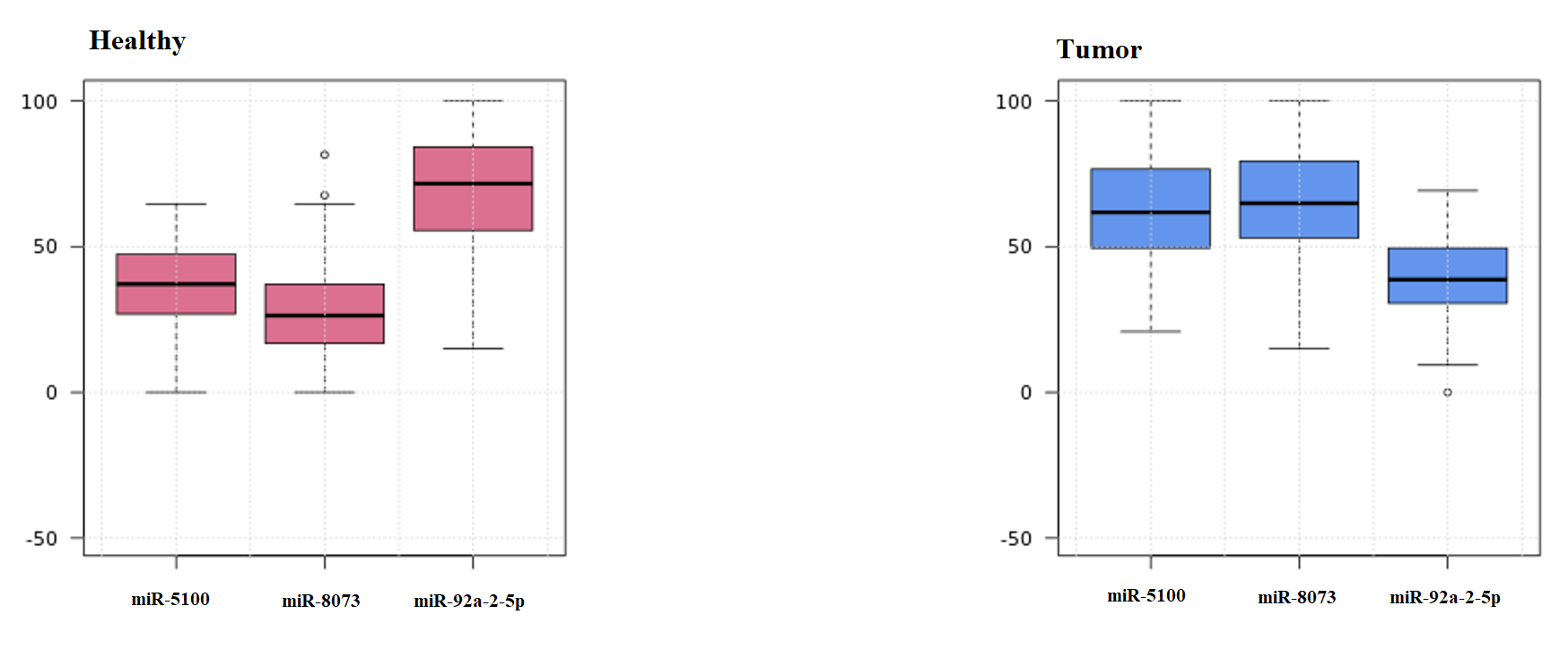


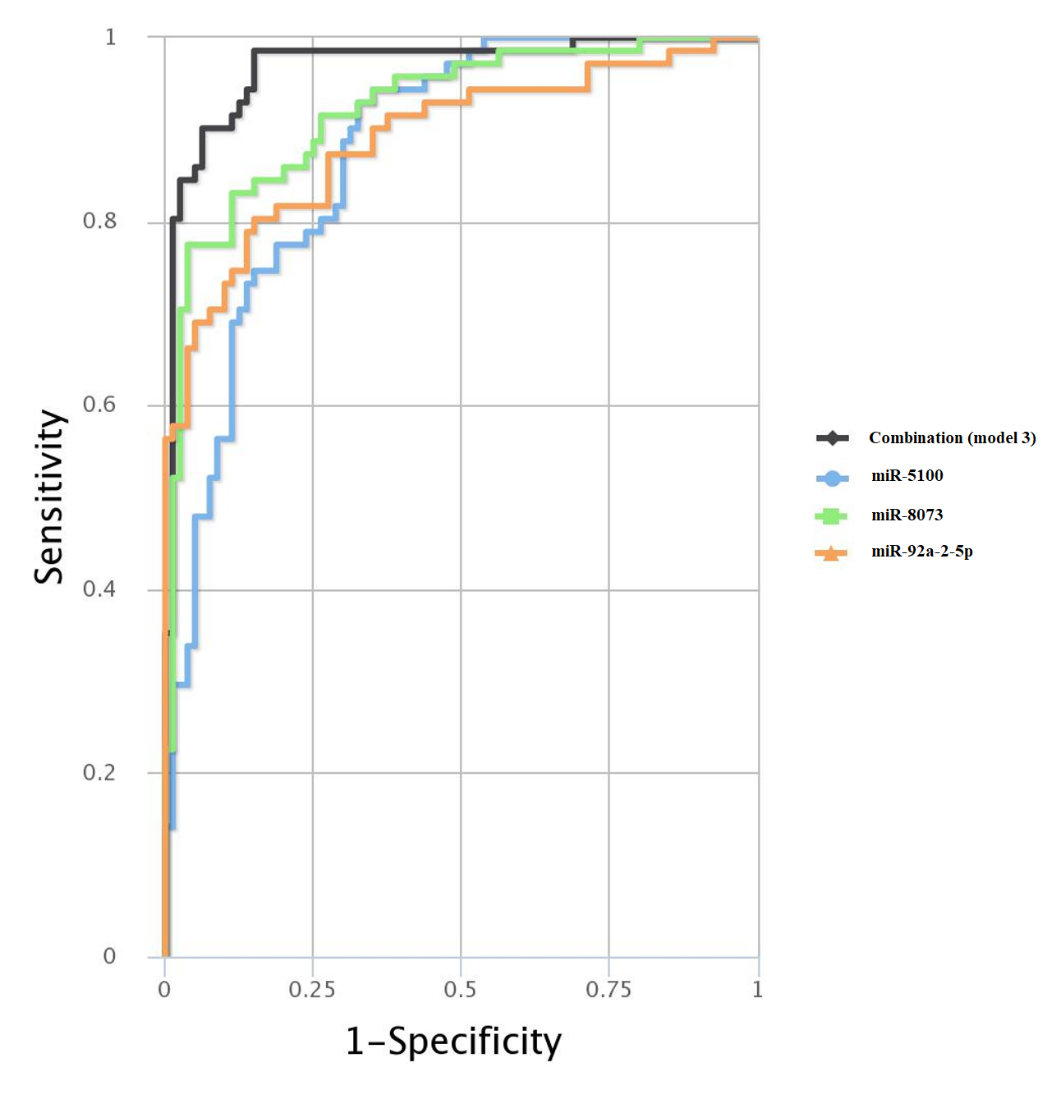


| **Symbol** | **AUC** | **SE** | **SP** | **Opt Cutoff** |
| --- | --- | --- | --- | --- |
| miR-5100 | 0.877 | 0.930 | 0.675 | 0.307 |
| Combination model 3 | 0.969 | 0.901 | 0.938 | 0.477 |
| miR-8073 | 0.923 | 0.775 | 0.962 | 0.644 |
| miR-92a-2-5p | 0.890 | 0.803 | 0.850 | 0.537 |

Predictions


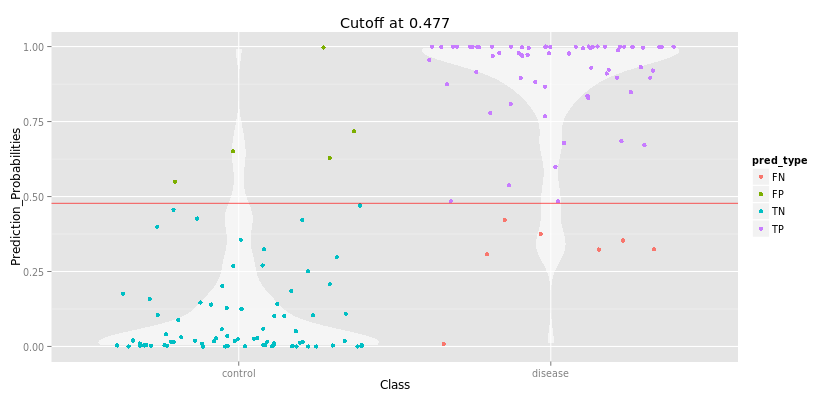


////////////////////////////////////////////////////////////////////////////////////////////////////////////////////////

Model 4


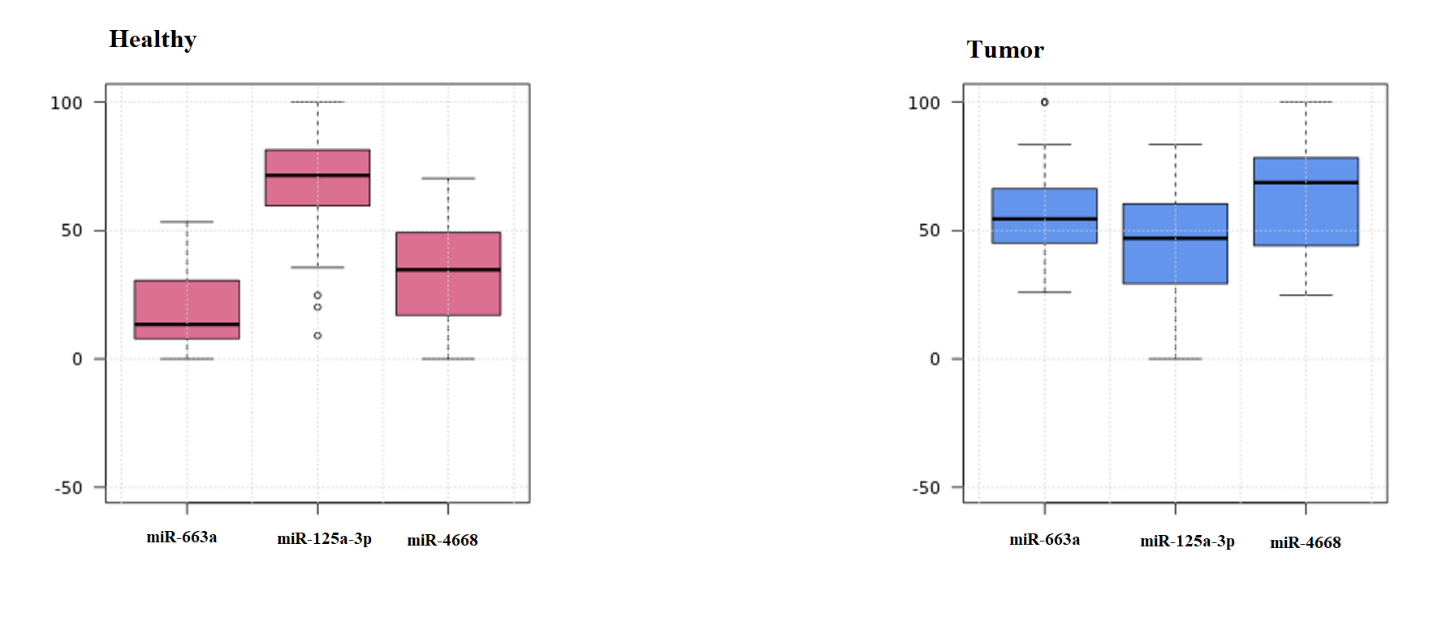


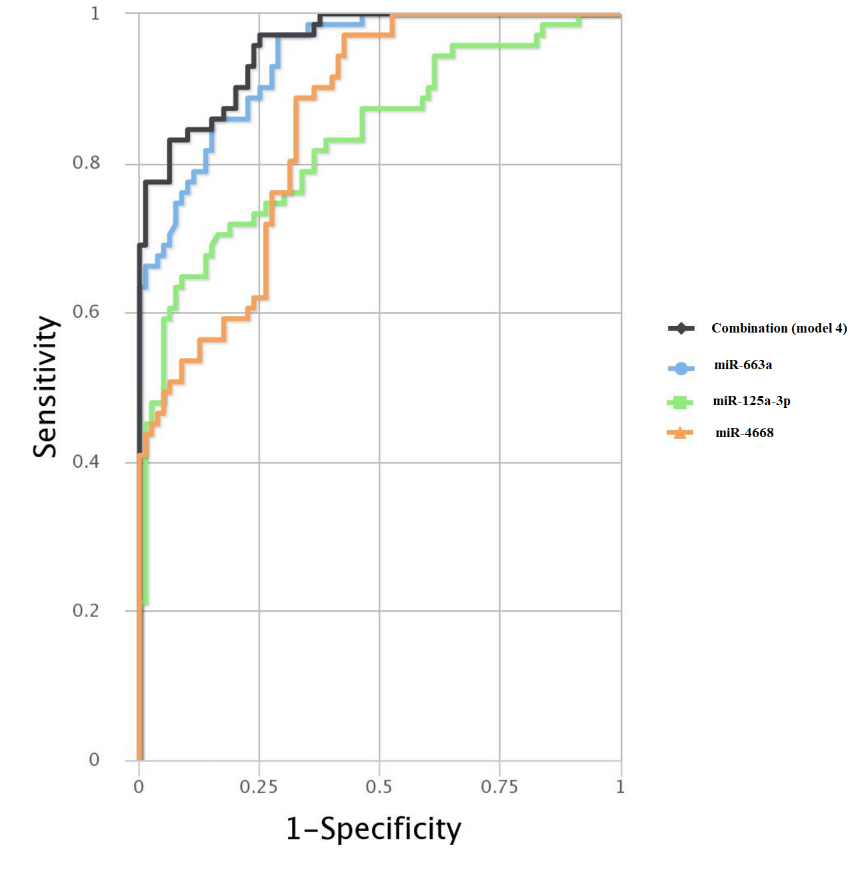


| **Symbol** | **AUC** | **SE** | **SP** | **Opt Cutoff** |
| --- | --- | --- | --- | --- |
| miR-663a | 0.939 | 0.859 | 0.850 | 0.354 |
| miR-125a-3p | 0.832 | 0.648 | 0.912 | 0.613 |
| miR-4668 | 0.852 | 0.887 | 0.675 | 0.306 |
| Combination model 4 | 0.957 | 0.831 | 0.938 | 0.593 |

Predictions


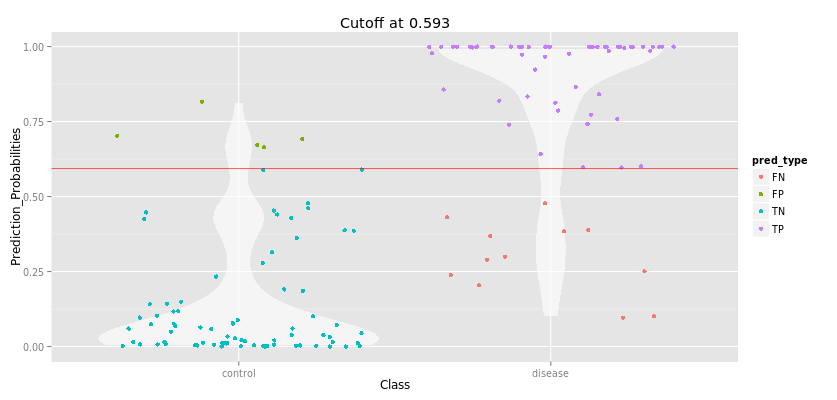


////////////////////////////////////////////////////////////////////////////////////////////////////////////////////////

Model 5


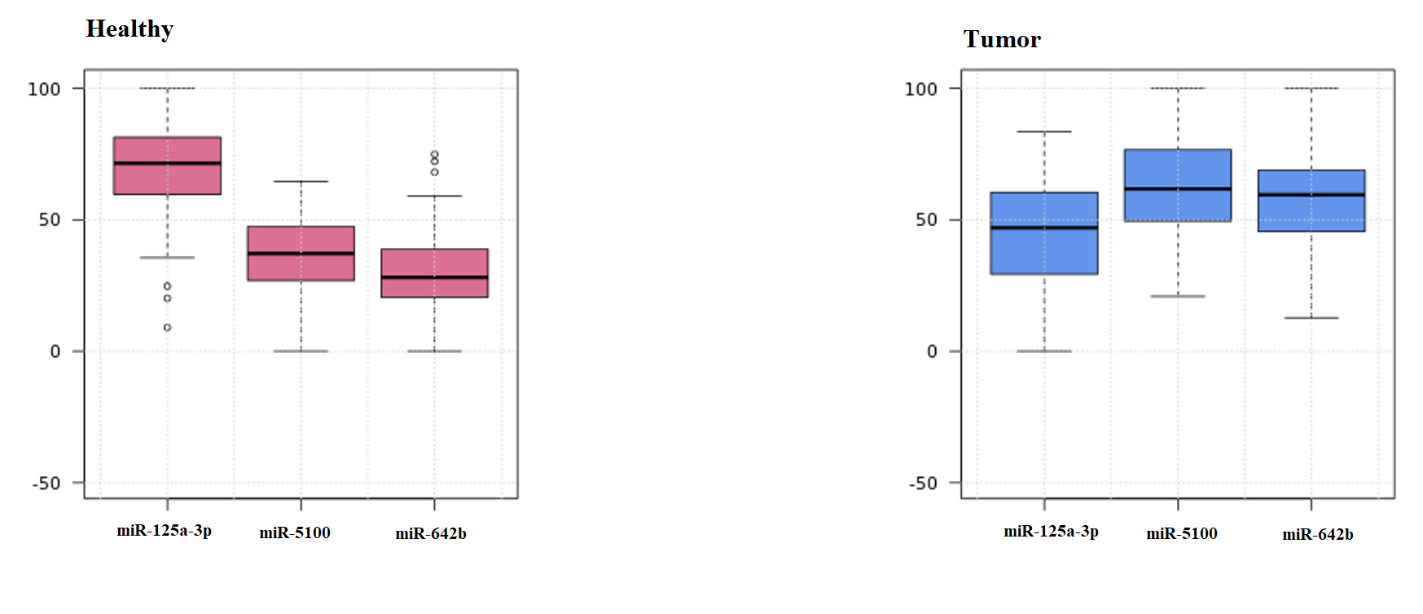


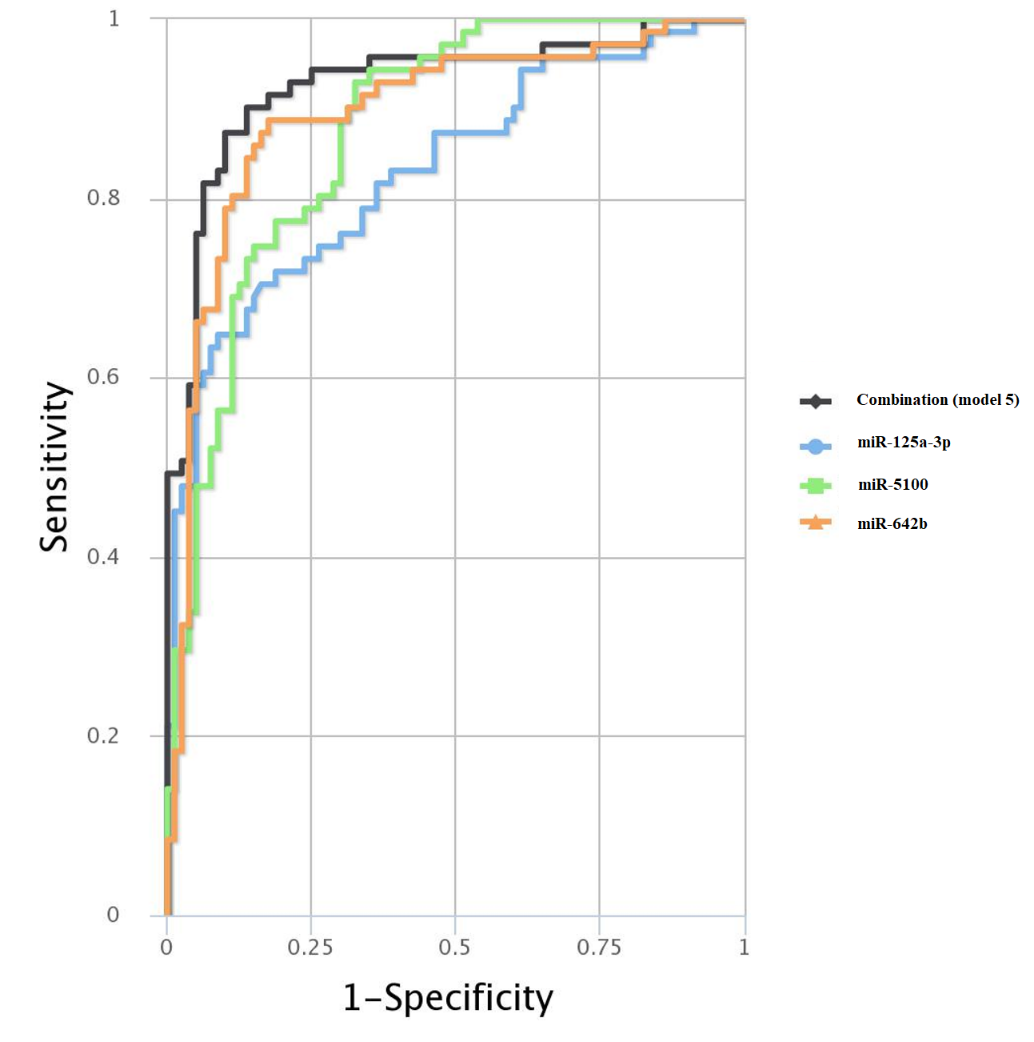


| **Symbol** | **AUC** | **SE** | **SP** | **Opt Cutoff** |
| --- | --- | --- | --- | --- |
| miR-125a-3p | 0.832 | 0.648 | 0.912 | 0.613 |
| Combination model 5 | 0.929 | 0.873 | 0.900 | 0.457 |
| miR-5100 | 0.877 | 0.930 | 0.675 | 0.307 |
| miR-642b | 0.895 | 0.887 | 0.825 | 0.403 |

Predictions


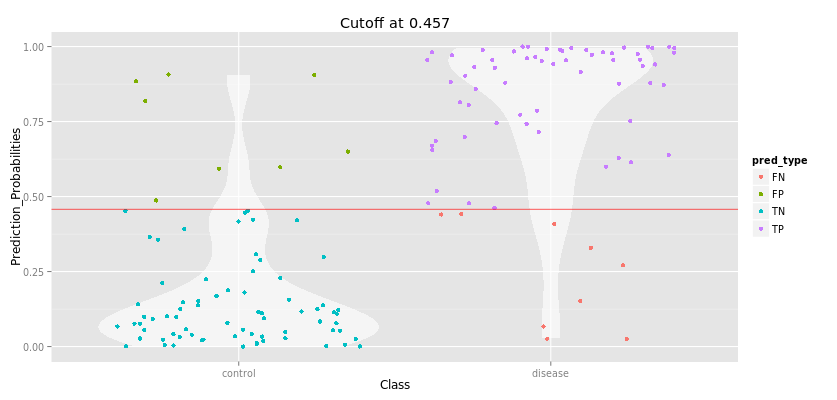


///////////////////////////////////////////////////////////////////////////////////////////////////////////////////////

**B**:

Kaplan Meier plots of considered miRNAs showing their relevance to survival time of the PC patients. Resulted from online database KM Plotter (<http://kmplot.com/analysis>). 6 out of 8 miRNAs from considered models, showed a significant association with the prognosis of the PC.


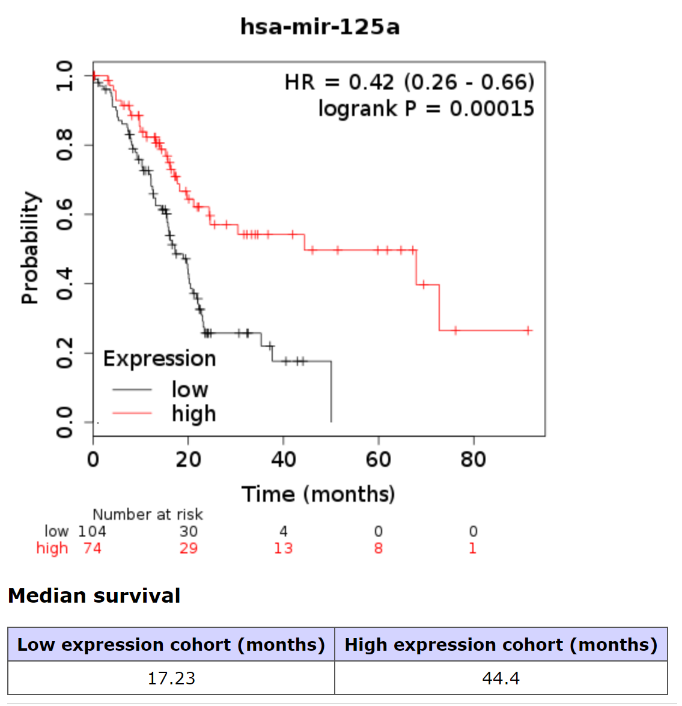

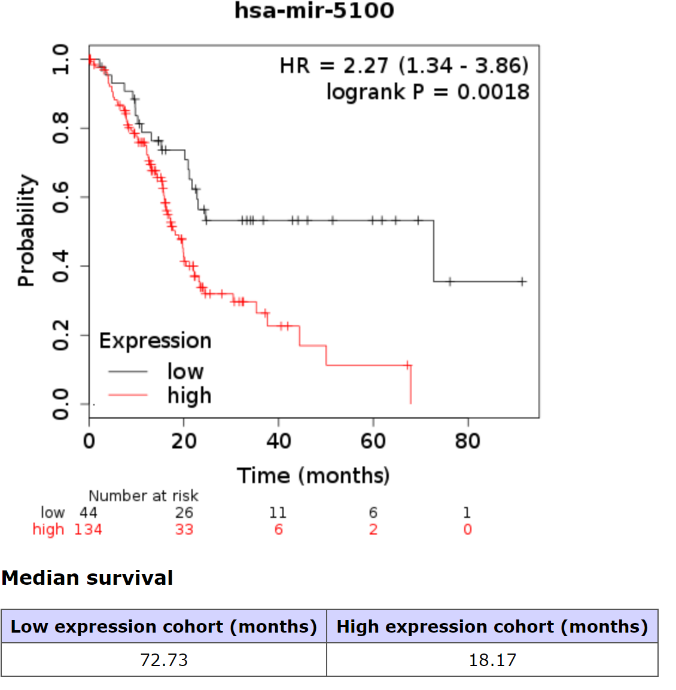


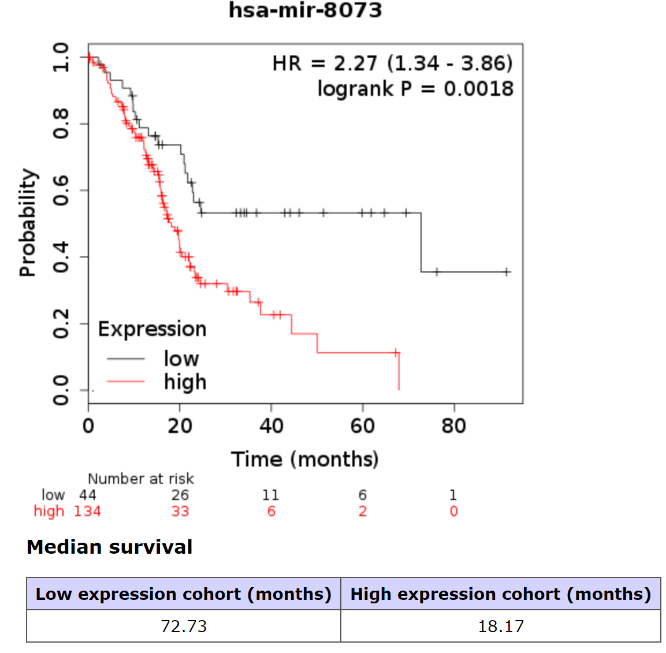

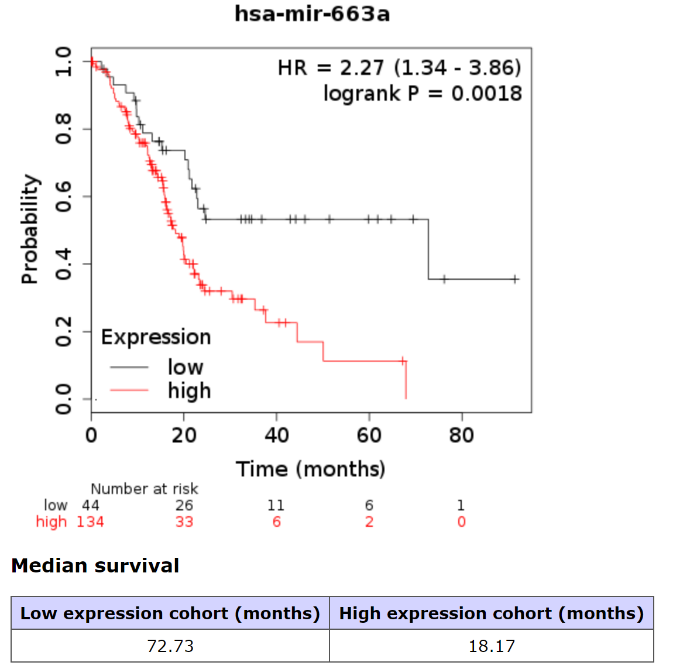


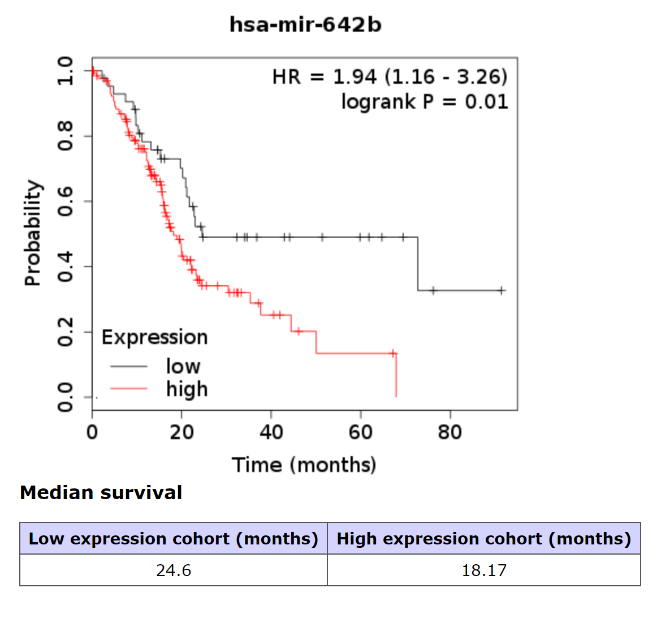

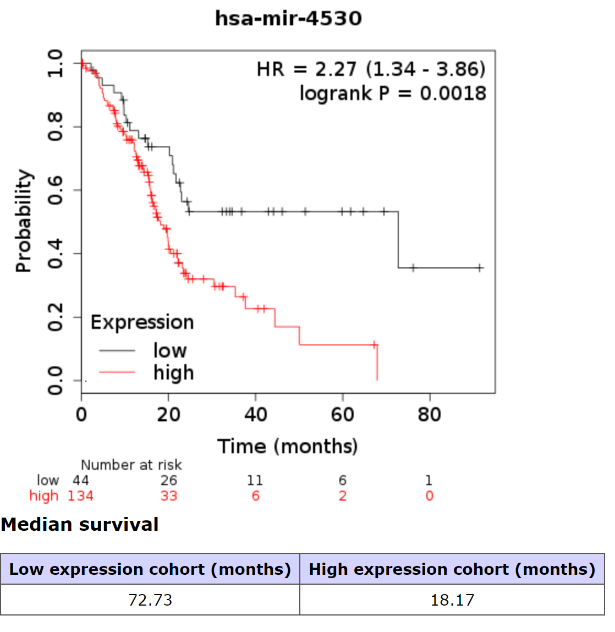

Supplement: Supplementary file 3 — Supplementary information 3. [file 41598_2020_64569_MOESM3_ESM.docx]
